# Supplementary material for: Quantitative analysis of relationship between mutation rate and speed of adaptation under antibiotic exposure in Escherichia coli
Source: PLoS Genet. 2025 Mar 28;21(3):e1011627. doi: 10.1371/journal.pgen.1011627 (PMC11975134; doi:10.1371/journal.pgen.1011627)
Supplement: S1 File — S1 Fig. Mutator Construction Procedure. Each box represents either a mutator strain or the wild-type strain. Arrows depict the lineage from parent to offspring in the construction of mutators. The list of mutations on the right details those shared between each parent-child pair (refer to S1 Table for the complete list of mutations). The values indicated in each strain box represent the growth rates (1/h; mean ± SD). S2 Fig. (A) Neutrality in mutation accumulation. The dN/dS ratio was calculated for each mutator strain and the wild-type strain. Error bars show the standard deviations between the MA lineages. (B) Relationship between growth rate and mutation rate. The horizontal error bars show the standard deviation across MA lineages, whereas the vertical error bars represent the standard deviation among replicate experiments in growth rate measurement. The sample sizes in the growth rate measurements were n=20 for wild-type strains and n=10 for mutant strains. The color and fill pattern of the markers correspond to those in Fig 3. S3 Fig. Experimental Evolution of Mutator Strains Under Antibiotics. For all combinations of drugs and strains, the changes in MIC over time are plotted. Each plot overlays data from four replicate series. Dashed lines represent the minimum and maximum MIC values attainable within the constraints of our experimental setup. S4 Fig. Reproducibility of the adaptation speed quantification. The MIC doubling rates were estimated by conducting independent experimental evolution trials with varying duration (9 days and 5 days, respectively). Each dot and error bar show the mean and standard deviation of MIC fold change per day, calculated from the data in S3 Fig. The black solid diagonal line means y=x identity line, while the blue line shows linear regression without intercept. The linear regression coefficient and corresponding R2 value were computed for these data points (N=13). S5 Fig. The relationship between the mutation rate, calculated b [file pgen.1011627.s001.docx]

**Supplemental Materials**

**Quantitative Analysis of Saturating Relationship between Mutation Rate and Speed of Adaptation under Antibiotic Exposure in *Escherichia coli***

Atsushi Shibai^1#^, Minako Izutsu^1,2,3,4#^, Hazuki Kotani^1^, Chikara Furusawa^1,5*^

^1^Center for Biosystems Dynamics Research, RIKEN, Osaka, Japan

^2^Department of Microbiology and Molecular Genetics, Michigan State University, Michigan, United States of America

^3^BEACON Center for the Study of Evolution in Action, Michigan State University, Michigan, United States of America

^4^Ecology, Evolutionary Biology and Behavior Program, Michigan State University, Michigan,

United States of America

^5^Universal Biology Institute, Graduate School of Science, The University of Tokyo, Tokyo, Japan


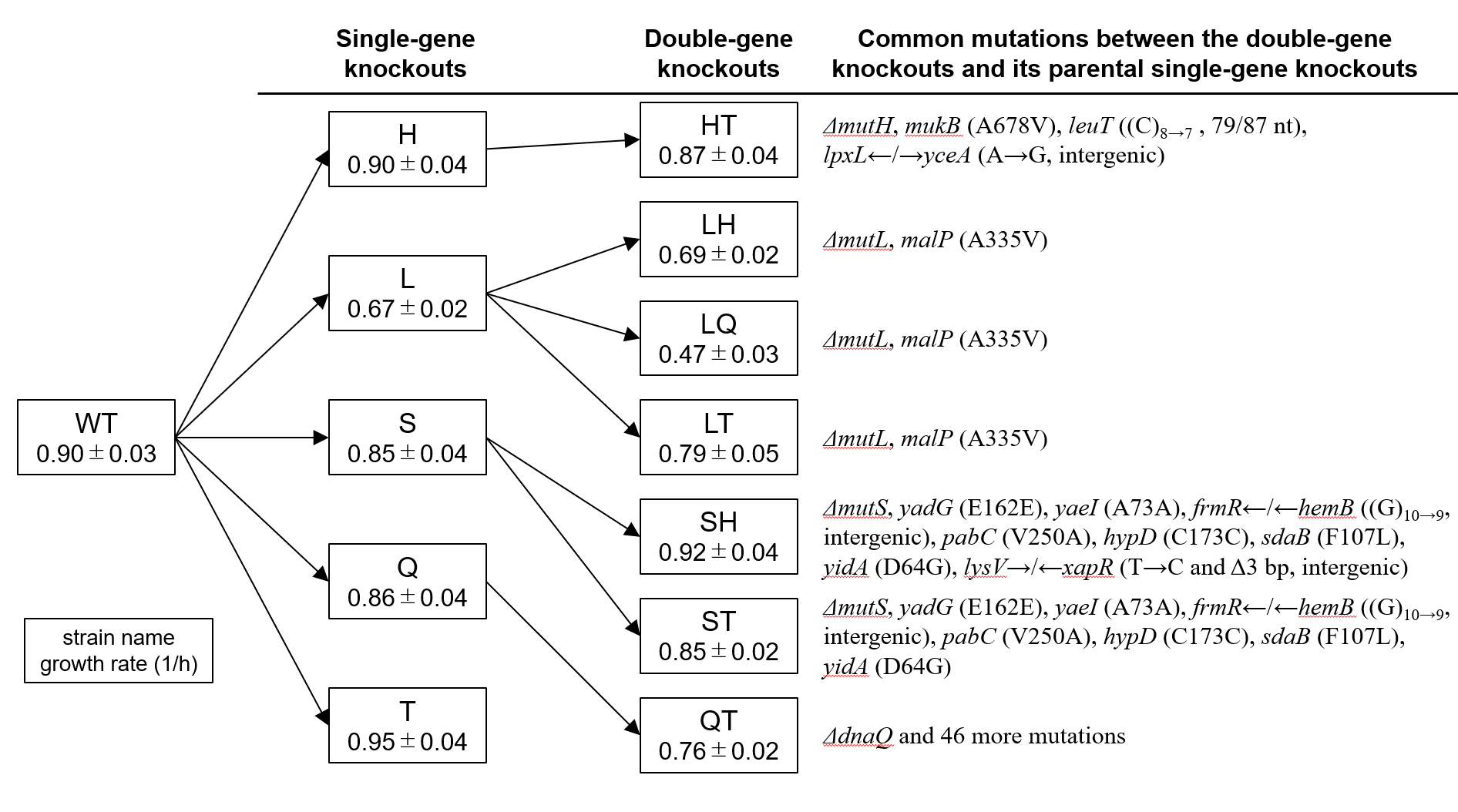


**Figure S1.** Mutator Construction Procedure. Each box represents either a mutator strain or the wild-type strain. Arrows depict the lineage from parent to offspring in the construction of mutators. The list of mutations on the right details those shared between each parent-child pair (refer to Table S1 for the complete list of mutations). The values indicated in each strain box represent the growth rates (1/h; mean ± SD).


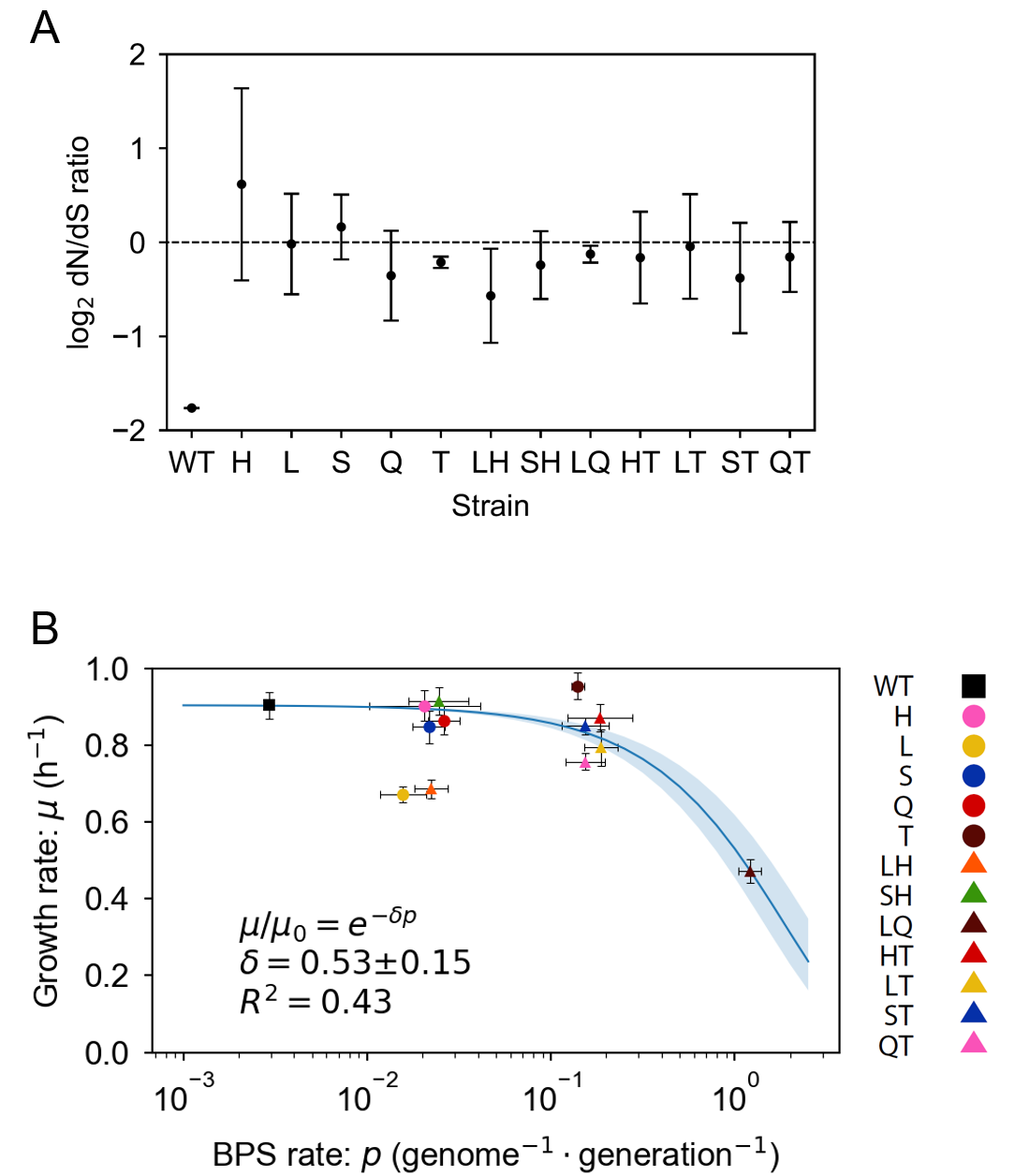


**Figure S2.** (A) Neutrality in mutation accumulation. The dN/dS ratio was calculated for each mutator strain and the wild-type strain. Error bars show the standard deviations between the MA lineages. (B) Relationship between growth rate and mutation rate. The horizontal error bars show the standard deviation across MA lineages, whereas the vertical error bars represent the standard deviation among replicate experiments in growth rate measurement. The sample sizes in the growth rate measurements were n=20 for wild-type strains and n=10 for mutant strains. The color and fill pattern of the markers correspond to those in Fig. 3.


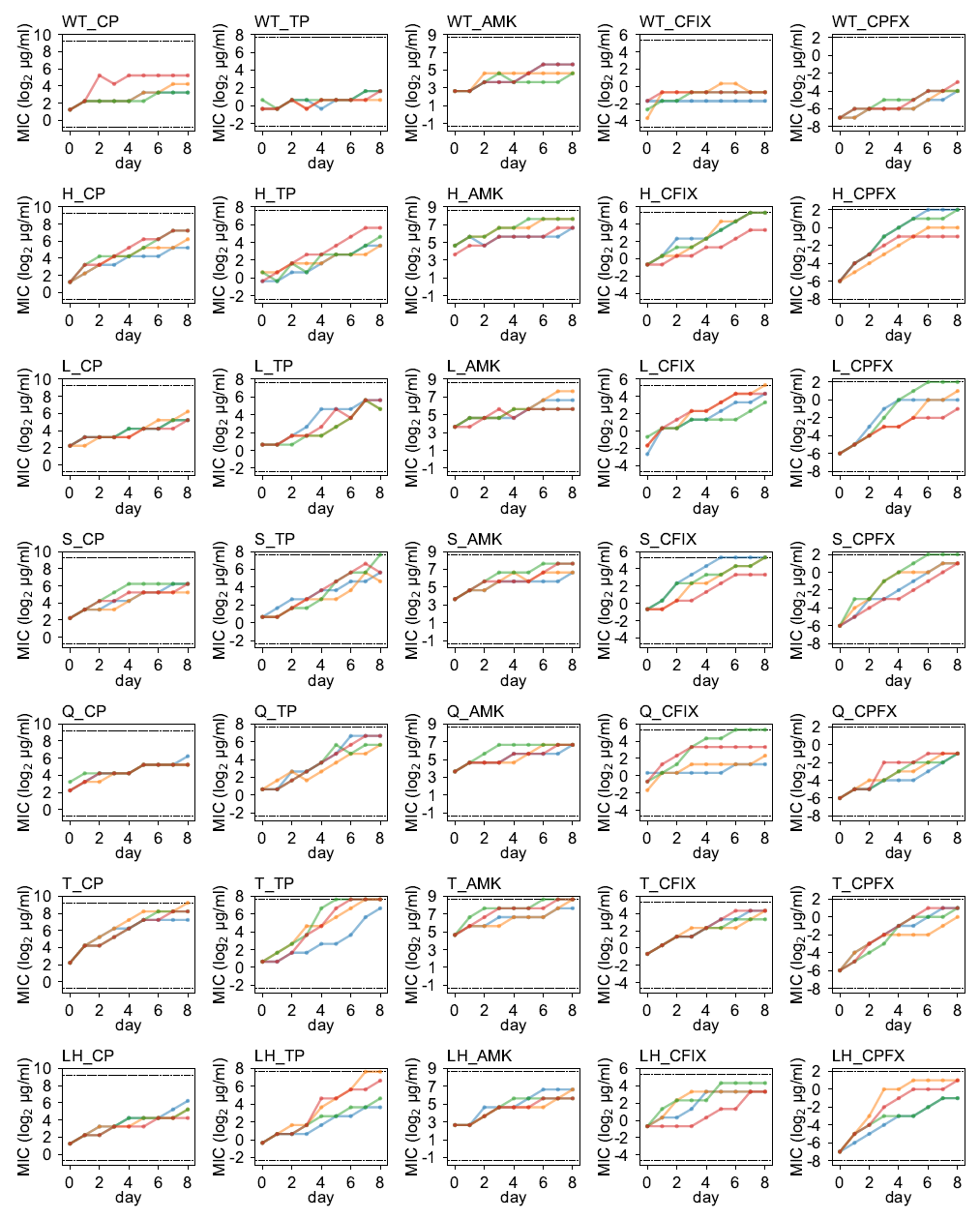


**Figure S3.** Experimental Evolution of Mutator Strains Under Antibiotics. For all combinations of drugs and strains, the changes in MIC over time are plotted. Each plot overlays data from four replicate series. Dashed lines represent the minimum and maximum MIC values attainable within the constraints of our experimental setup.


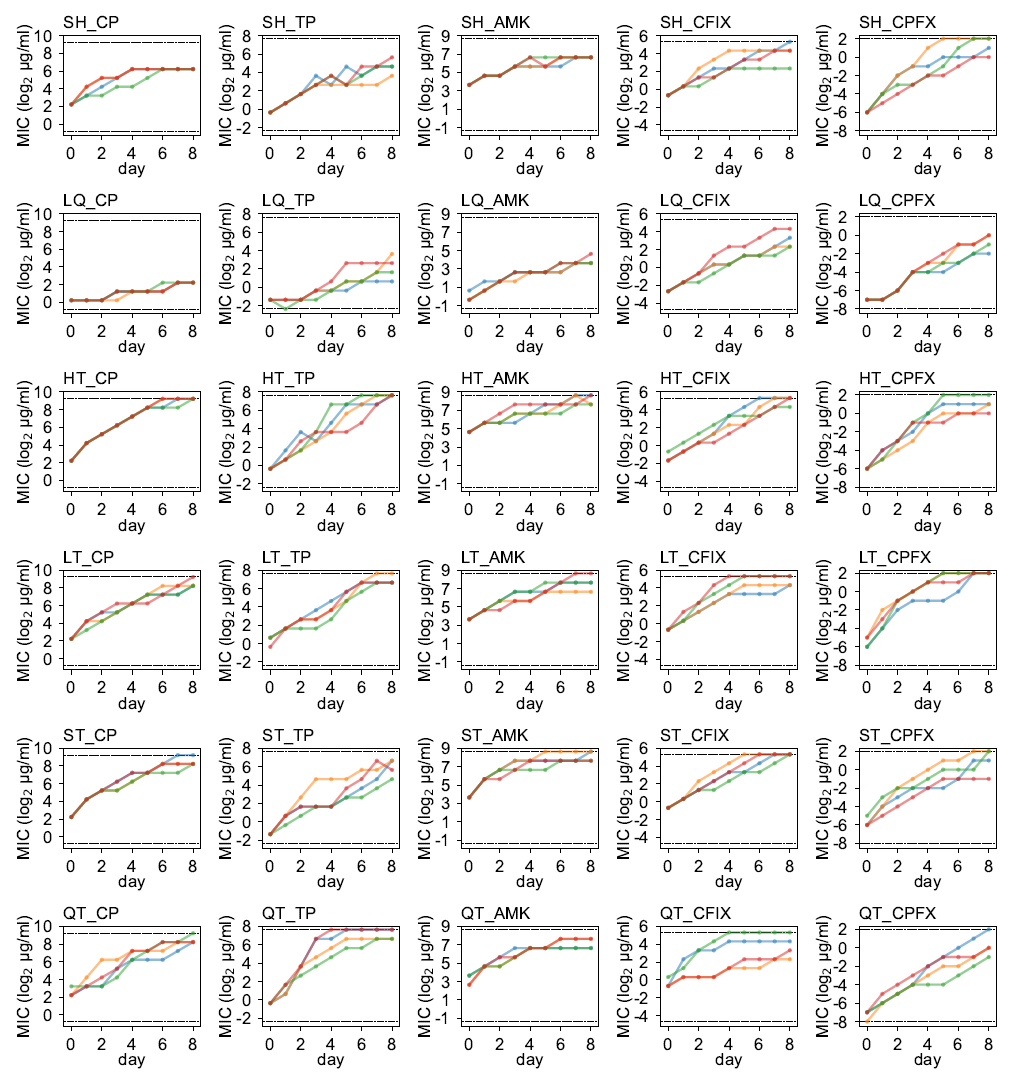


**Figure S3.** (continued)


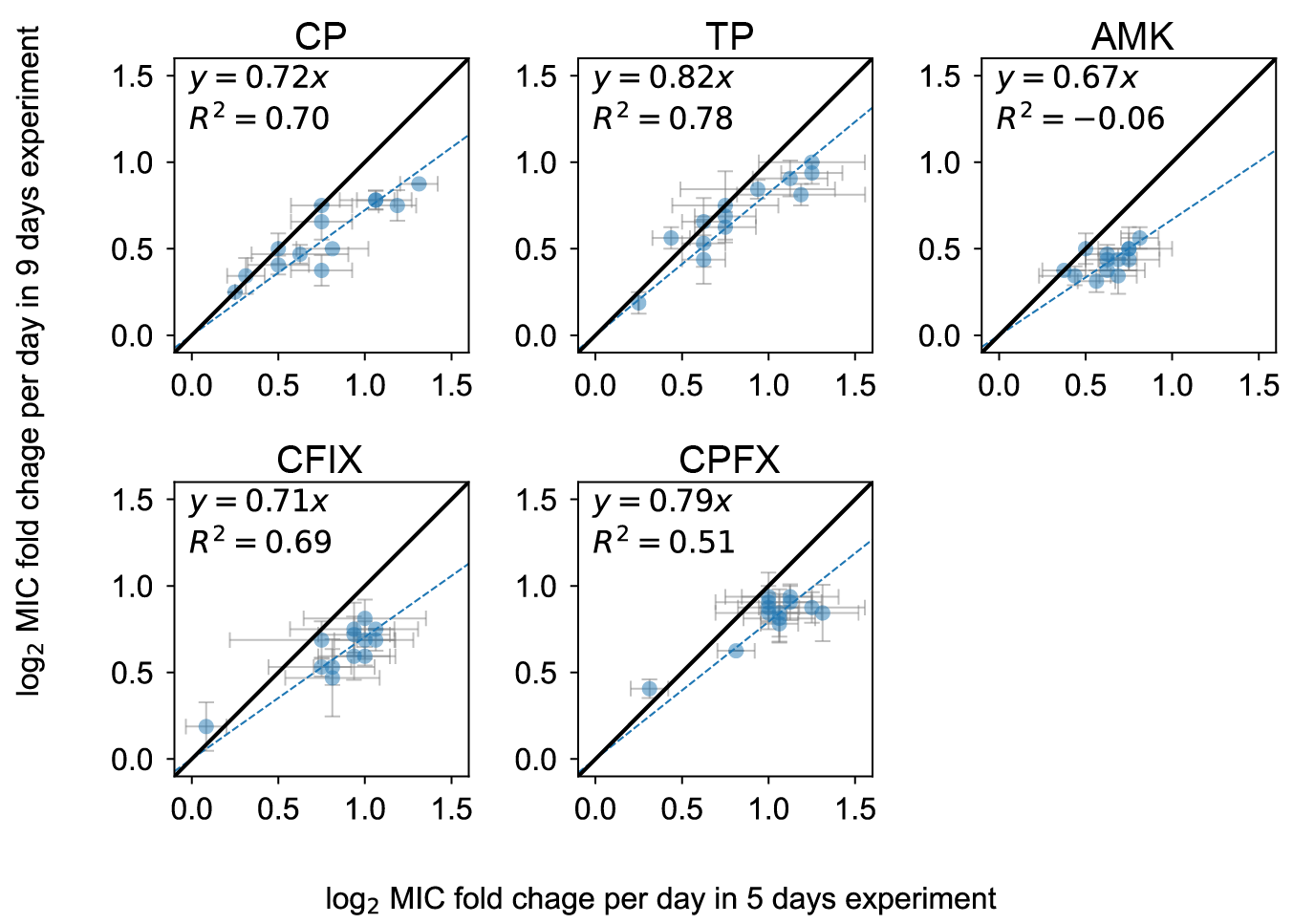


**Figure S4.** Reproducibility of the adaptation speed quantification. The MIC doubling rates were estimated by conducting independent experimental evolution trials with varying duration (9 days and 5 days, respectively). Each dot and error bar show the mean and standard deviation of MIC fold change per day, calculated from the data in Fig. S3. The black solid diagonal line means y=x identity line, while the blue line shows linear regression without intercept. The linear regression coefficient and corresponding R2 value were computed for these data points (N=13).


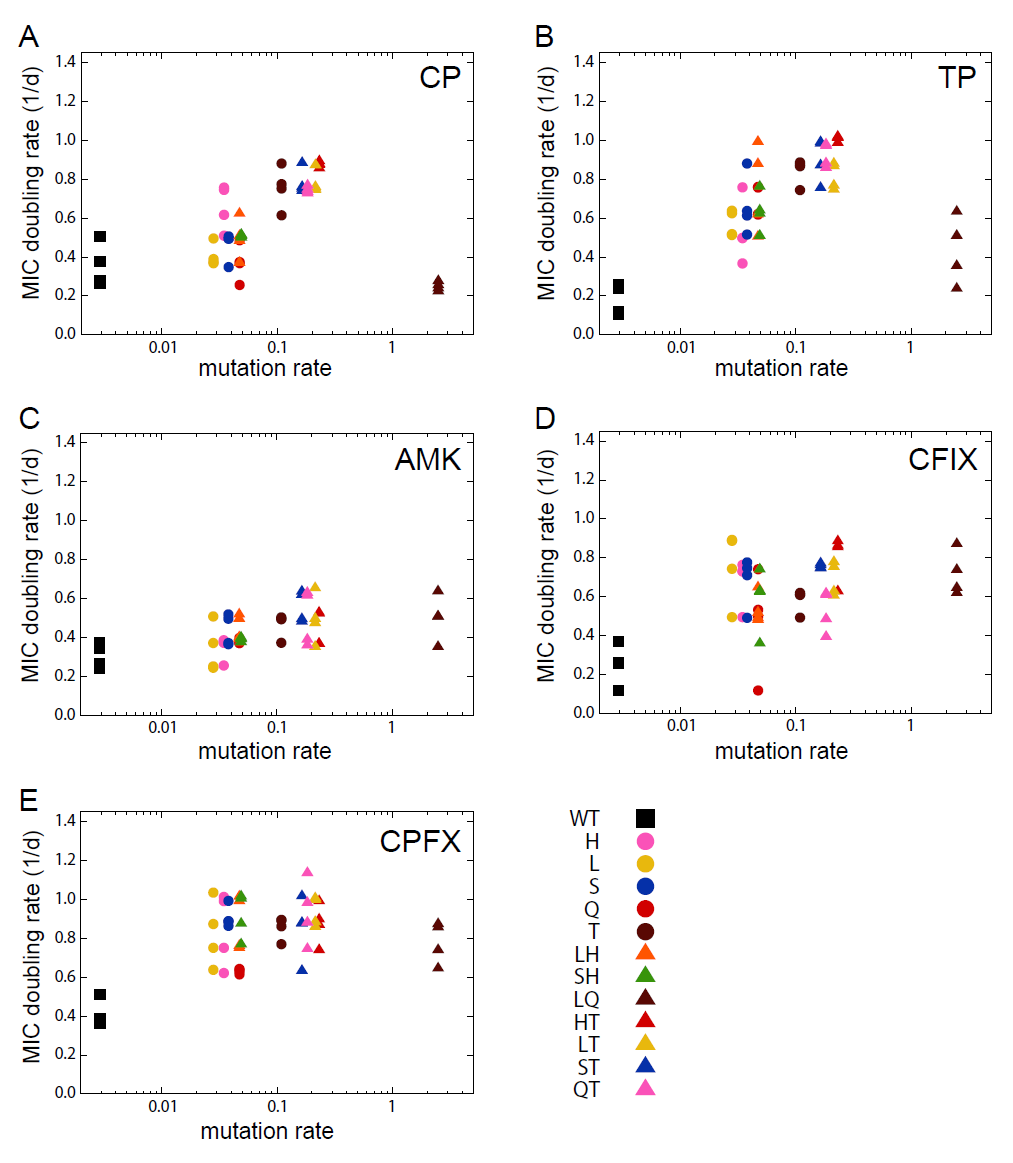


**Figure S5.** The relationship between the mutation rate, calculated by the sum of fixed SNPs and indels, and the MIC doubling rate is shown. Each dot represents an experimental observation from 13 strains across four replicate serial transfer cultures. To prevent overlap of data points, small Gaussian noise (mean = 0, standard deviation = 0.05) was added to the y-coordinates.


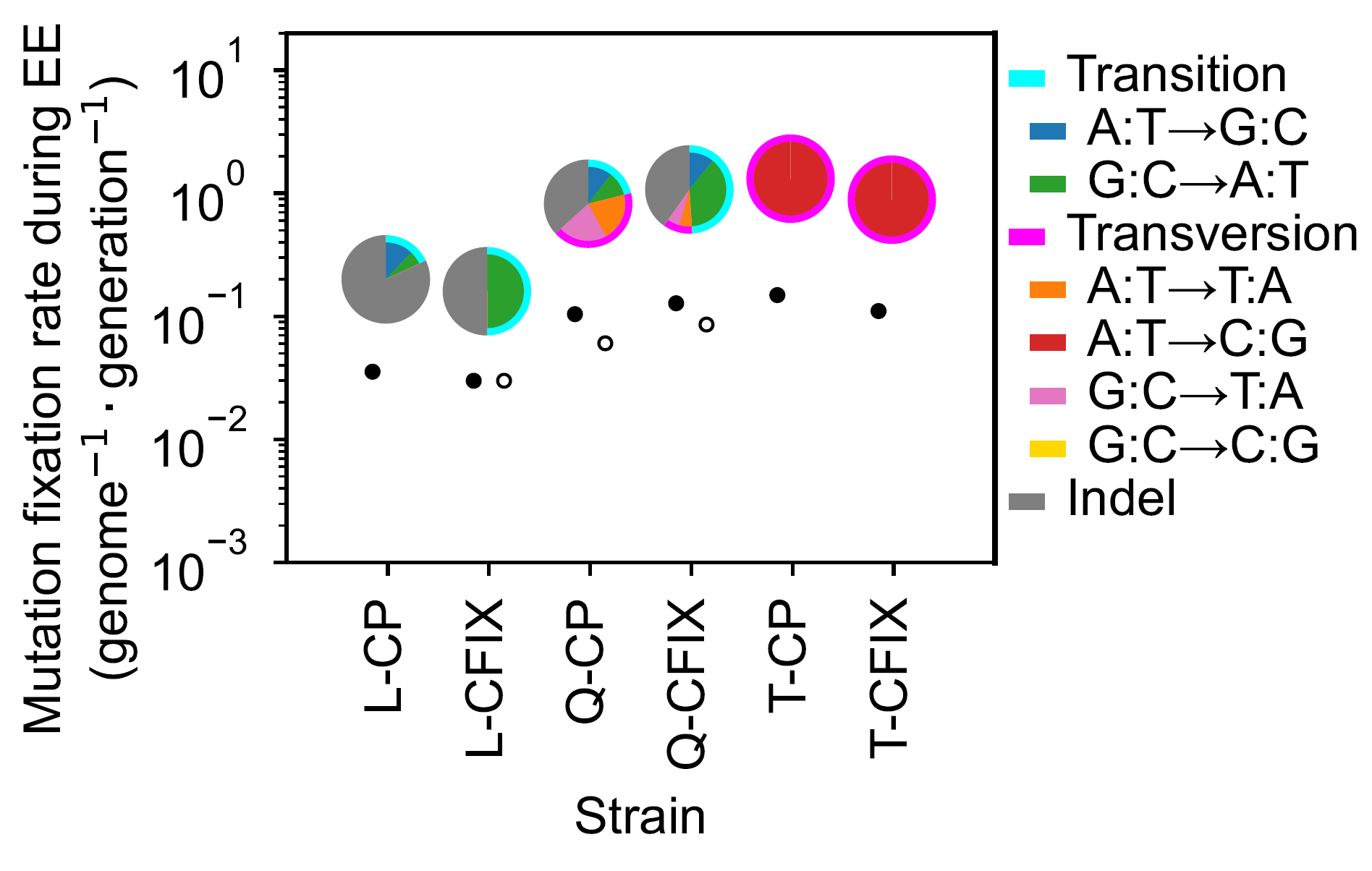


**Figure S6.** Mutation Spectra in Resistant Strains. The distribution of substitution patterns and indels calculated by 24 resistant strains (three ancestor mutators, two antibiotics, and four replicates) is presented. Each dot represents the mutation fixation rate during the experimental evolution calculated based on SNPs (closed circle) or indels (open circle).


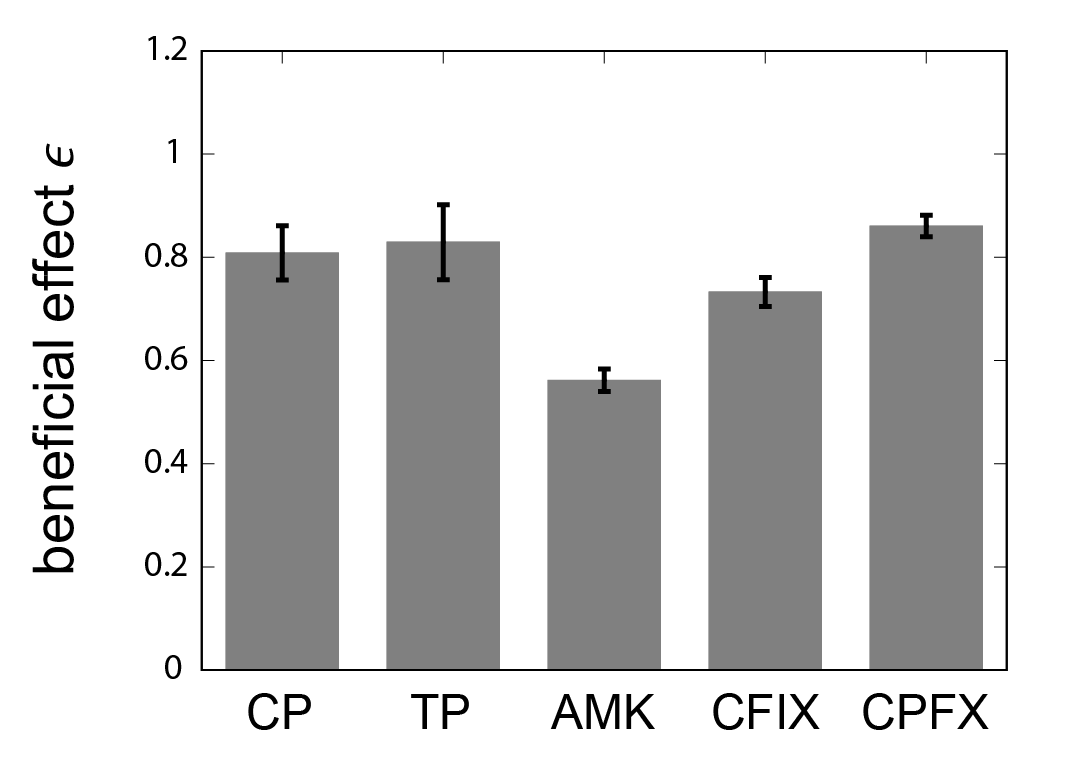


**Figure S7.** Estimated parameter $\epsilon$ representing the beneficial effect of each mutation. The mean value estimated through 100 bootstrap resampling is represented by the bars, while the error bars represent the standard error.


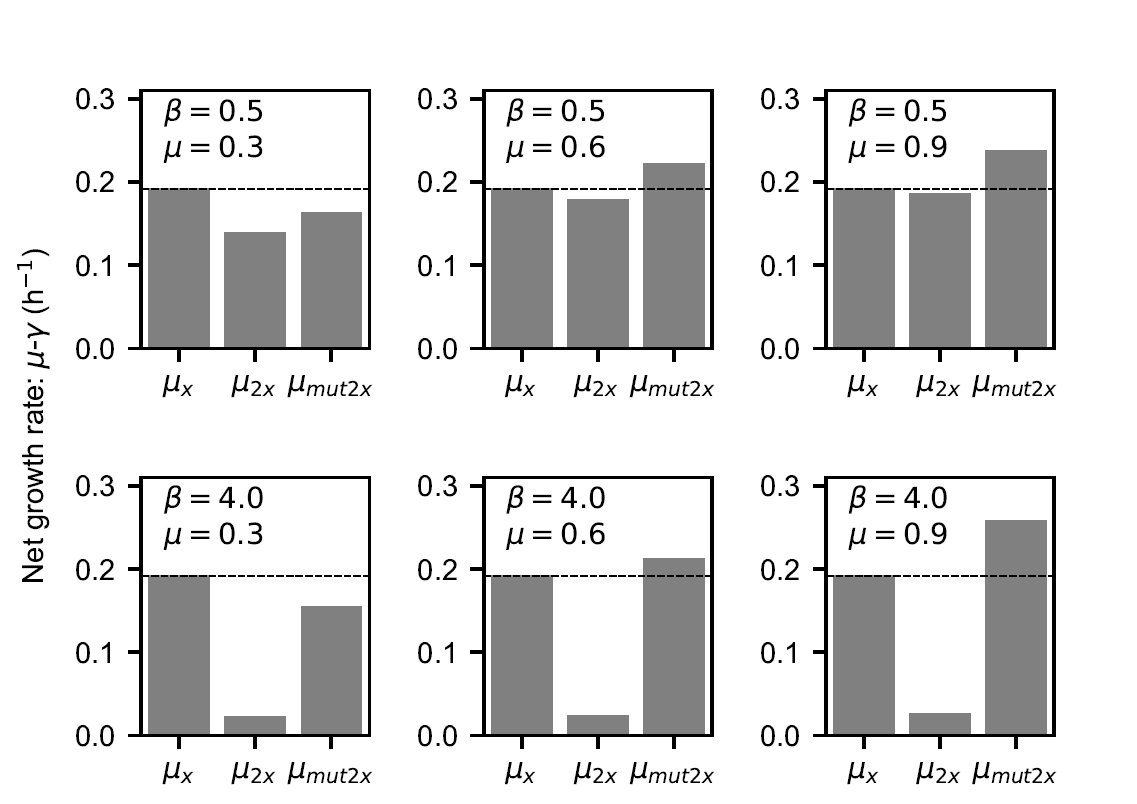


**Figure S8.** A doubled drug concentration with large β remarkably reduces the growth rate. We defined the net growth rate as μ-γ where μ is the drug-free growth rate and γ is the death rate by drug treatment. $\mu_{x}$ is the net growth rate with drug concentration $x$, $\mu_{2x}$ is the net growth rate with drug concentration $2x$, and $\mu_{mut2x}$ is the net growth rate with drug concentration $2x$ for cells with one mutation of beneficial effect $\epsilon$. We assume here a situation where the growth rate and dilution rate are balancing at the drug concentration $x$, i.e. $\mu_{x}=\ln100/24 \cong0.19 [h^{-1}]$. The black dashed line represents $\mu_{x}=0.19$. We used $\epsilon=0.9$ and $b_{i}=1$ for these simulations.

**
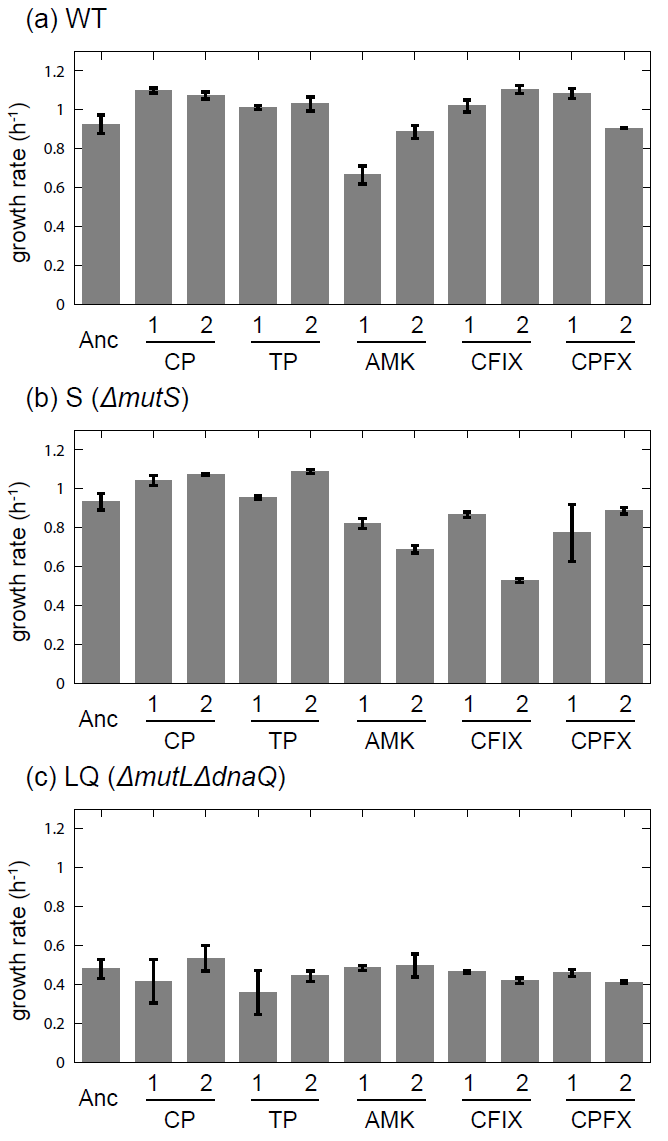
**

**Figure S9.** Changes in growth rate during resistance evolution. Growth rates for three strains (WT, S, and LQ) were quantified both before and after experimental evolution. For each selection drug, two independently evolved strains were analyzed. The bars represent the standard deviation of three replicate measurements.


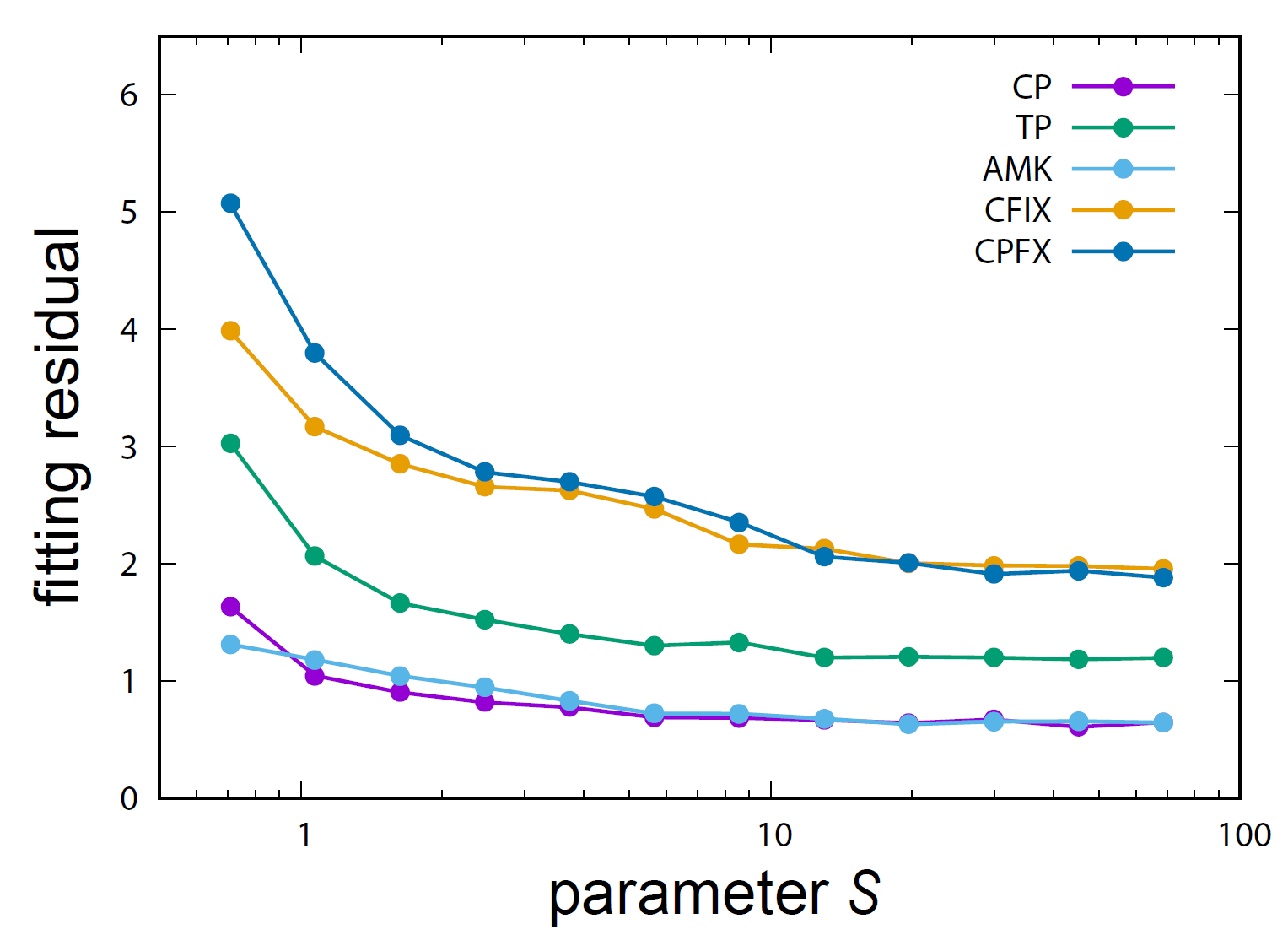


**Figure S10.** Relationship between the parameter $S$ and the residual of fitting. The parameter $S$, which describes the saturation effect of mutation accumulation in the form $b_{i}=b_{0}+{i\epsilon S}/{(i+S)}$, is plotted against the residual of the fitting. The modified model incorporating the saturation effect was used to fit the experimental data shown in Fig. 3. As observed, the residuals decrease with increasing $S$, suggesting that the saturation effect is negligible.
