# Supplementary material for: Quantitative analysis of relationship between mutation rate and speed of adaptation under antibiotic exposure in Escherichia coli
Source: PLoS Genet. 2025 Mar 28;21(3):e1011627. doi: 10.1371/journal.pgen.1011627 (PMC11975134; doi:10.1371/journal.pgen.1011627)
Supplement: Attachment — Submitted filename: Response_to_Reviewers.pdf [file pgen.1011627.s012.pdf]

Reply to Editor

We would like to thank the Editor for the constructive suggestion to improve our manuscript. We have revised the manuscript following the suggestion. Our response to the comment is shown below.

Comment 1: However, these new results deserve further discussion and contextualization. In particular, the increased indel frequency is primarily observed in the lower tier mutators and the LQ outlier, while indels are considerably rare in the higher tier mutators. As indels are expected to result in larger effects on average compared to base pair substitutions, and resistance mutations often arise in essential genes where disruption by indels can be lethal, it is important to consider the implications of the differing mutation spectra on your results.

Response 1: As the editor noted, indels and base-pair substitutions (BPSs) have distinct impacts on phenotype, and variations in their frequencies may substantially influence the evolutionary process. We agree that integrating this perspective into the discussion enhances the significance of this study. In response, we have incorporated the following paragraph into the revised manuscript to address this point.

Revision 1 (Line 453):
The analysis of the mutational spectrum also revealed that the ratio of indels to BPSs varied depending on the knockout gene(s). As shown in Fig. 1, when the LQ strain is excluded as an outlier, mutator strains with a high fixation rate of BPSs do not necessarily exhibit a correspondingly high frequency of indels. Given that indels often have a more pronounced impact on phenotypic changes compared to BPSs and are more likely to result in lethal mutations, substantial variation in the ratio of indels to BPSs across different mutation rates may influence the evolutionary dynamics of distinct mutator strains. Given the limited amount of data and the challenges associated with conducting statistical analyses, it is difficult to draw definitive conclusions from this study. Future research with sufficient data should be conducted to further explore the relationship between the mutational spectrum in mutators and the evolution of drug resistance.

Reply to Reviewer #1

We would like to thank the reviewer for the constructive suggestions to improve our manuscript. We have revised the manuscript following these suggestions. Our responses to specific comments are shown below.

Comment 1: The authors have satisfactorily addressed most of my specific comments but my overall opinion of this paper is not changed significantly by the revisions. While the results are interesting, the authors' interpretations rely heavily on the behavior of one strain, the mutL dnaQ double mutant (LQ). Indeed, the relationship between mutation rates and the increases in MIC of the strains in Figure 3 can be interpreted in every case as linear with one clear outlier, LQ. Likewise, the relationship between mutation rates and growth rates of the strains in Fig. S2B can be interpreted as a straight line, no effect, with LQ again being a clear outlier and L and LH also lying below the line. All three original reviewers brought this up; reviewer #3 was most articulate, pointing out the possibility of "synthetically deleterious combination of these alleles". The results in Fig. S2B verify this possibility. The authors have addressed this interpretation in the text, justifying their modeling by with the assumption that the effects of any detrimental factors are proportional to the effects of the observed mutation rates. But this assumption has no evidential support. Factors that reduce growth rates may directly or indirectly also reduce mutation rates and/or the rates of MIC increase. These considerations cloud the interpretation of the results. I feel that most readers, if they read carefully, will come to the same conclusion.

Response 1-1: We acknowledge the reviewer's comment that the observed non-monotonic relationship between mutation rate and evolutionary adaptation rate is based on a single data point (the LQ strain) and that, if this point is excluded, the relationship can be approximated as linear. In response, we have modified the manuscript title to remove the term "saturating", and we have revised the Abstract, Introduction, Results, and Discussion sections accordingly as shown below. Additionally, in the first paragraph of the Discussion section, we emphasize the significance of the LQ strain as an exception to the general trend. This data point highlights that the relationship between mutation rate and evolutionary adaptation rate is potentially non-monotonic under certain conditions, warranting further investigation.

Revision 1-1-1 (Abstract):
Mutations are the ultimate source of biological evolution that creates genetic variation in populations. Mutations can create new advantageous traits but can also potentially interfere with pre-existing organismal functions. Therefore, organisms may have evolved mutation rates to appropriate levels to maintain or improve their fitness. In this study, we aimed to experimentally quantify the relationship between the mutation rate and evolution of antibiotic resistance. We conducted an evolution experiment using 12 *Escherichia coli* mutator strains with increased mutation rates and five antibiotics. Our results demonstrated that the rate of adaptation generally increased with higher mutation rates, except in a single mutator strain with the highest mutation rate, which exhibited a significant decline in evolutionary adaptation speed. To further elucidate these findings, we developed a simple population dynamics model that successfully recapitulated the observed dependence of adaptation speed on mutation rate. These findings provide important insights into the evolution of mutation rate accompanied by the evolution.

Revision 1-1-2 (Introduction; Line 111):
The results revealed that the speed of adaptation, as measured by the rate of increase in the minimum inhibitory concentration (MIC), increased approximately linearly with mutation rate, except for a single mutator strain with the highest mutation rate, which exhibited a significant decrease in adaptation speed. Additionally, the results demonstrated that this

dependency varied between bacteriostatic and bactericidal antibiotics. We successfully reproduced these mutation-rate dependencies using numerical simulations of a population dynamics model. These findings offer important insights into the evolution of mutation rates accompanying evolutionary processes.

Revision 1-1-3 (Results; Line 203):
Figure 3 illustrates the relationship between the mutation rate and the rate of adaptation for each antibiotic. In this analysis, the mutation rate was calculated based on the frequency of SNPs. As observed, the rate of evolutionary adaptation generally increased with mutation rate, except for the LQ strain, which had the highest mutation rate and often exhibited significantly slower adaptation speeds. A similar pattern is also observed when the mutation rate is calculated using the combined frequency of BPSs and indels (Fig. S5).

Revision 1-1-4 (Discussion; Line 360):
In this study, we conducted quantitative analysis to investigate how changes in mutation rates affect the evolution of antibiotic resistance in *E. coli*. To achieve this goal, we generated a library of *E. coli* mutator strains using single and double deletions of genes related to DNA replication and error correction. We quantified the mutation rates of these mutators using MA experiments, which resulted in a range of mutation rates caused by gene deletions. We then performed evolution experiments starting from these mutators by serial transfer culture under five antibiotics and quantified the rate of MIC increase over time. The results demonstrated that, for nearly all mutator strains, the rate of adaptation positively correlated with mutation rate. However, an exception was observed in the LQ strain, which exhibited the highest mutation rate but frequently showed a significant reduction in the adaptation rate (Fig. 3). This finding highlights that the relationship between mutation rate and evolutionary adaptation rate is potentially non-monotonic under certain conditions.

Response 1-2: There are two possible explanations for the significant decrease in the growth rate of the LQ strain. One is the synergistic effect of *mutL* and *dnaQ* deletions, as suggested by the reviewers; the other is the additional impact of deleterious mutations, as proposed in our model. As the reviewer pointed out, we do not have conclusive data to distinguish between these possibilities, and both appear equally plausible.

Nevertheless, our population dynamics model, which assumes the accumulation of deleterious mutations, provides a general framework for describing the relationship between growth inhibition and evolutionary rate. This approach may serve as a useful method for characterizing the interplay between mutation rate and evolutionary adaptation rate, which we believe adds value to the study.

Furthermore, previous studies examining the relationship between mutation rate and growth rate across various mutator strains and culture conditions have consistently demonstrated that an elevated mutation rate frequently leads to a decline in growth rate (Ishizawa et al., Genes to Cells, 20(1), 68-76, 2015; see the figure below). These observations suggest that the cumulative effects of deleterious mutations may contribute to the observed growth defects.

To appropriately incorporate these discussions, we have revised the Discussion section as follows.

Revision 1-2 (Discussion; Line 372):
The observed reduction in the adaptation rate of the LQ strain is associated with its growth defect (Fig. S2B). However, the underlying mechanism responsible for this growth defect remains unclear. One possible explanation is that the simultaneous deletion of the *mutL* and *dnaQ* genes exerts a synthetic effect, thereby impairing growth. Alternatively, this study

posits a model in which the accumulation of deleterious mutations and other detrimental effects, proportional to the mutation rate, ultimately leads to a reduction in growth rate. Both hypotheses are equally plausible and provide potential explanations for the observed decrease in the evolutionary rate due to growth impairment. However, no experimental data is currently available to distinguish between these two possibilities.

Nevertheless, our population dynamics model, which assumes the accumulation of deleterious mutations, provides a general framework for describing the relationship between growth inhibition and evolutionary rate. This approach may offer a suitable method for characterizing the interplay between mutation rate and evolutionary adaptation rate. Furthermore, previous studies investigating the relationship between mutation rate and growth rate across various mutator strains and culture conditions have consistently demonstrated that an elevated mutation rate frequently results in a decline in growth rate [34]. These observations suggest that a spectrum of deleterious mutations may collectively contribute to the observed growth defects.

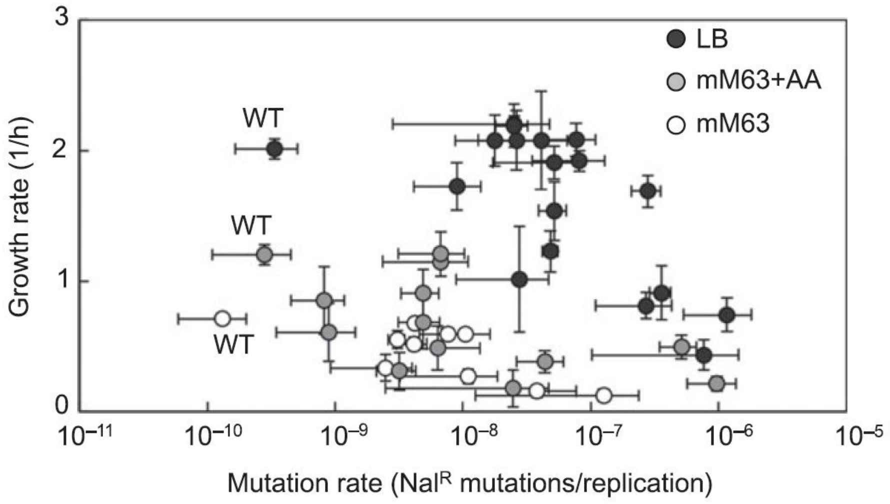

Figure 2 in Ishizawa et al. Relationship between mutation rates and growth rates. In this study, mutator strains were created using single and double deletion of *mut* genes.

Comment 2: The authors repeatedly attribute the growth defects of the LQ strain to hyperactivation of mismatch repair (MMR) and reference Stefan et al, Ref. 35, for this possibility. But, they have misinterpreted Ref. 35. In Stefan et al, MMR is not inferred to affect the growth rate, but to act to keep the mutation rate low when cell growth is slow due to the silencing of dnaQ. They conclude: “the ε subunit primarily affects the processivity of DNA polymerase III” and that is what causes the growth defects.
The authors also reference a paper by Iyer et al, 2020, showing that overexpression of MutS causes double-strand breaks and inhibits cell division. However, these effects were with MutS dramatically overinduced. At lower levels (10x normal) MutS overexpression had no growth effect and, in fact, elevated some mutation rates, which effect was suppressed by overexpression of MutL. Also, as far as this reviewer can find, this paper did not appear in a peer-reviewed journal. Regarding the new genetic information, I am surprised the authors did not make a little more about the information. Specifically, the fact that 'expected' mutations in the canonical resistance target, gyrA, were found in the ciprofloxacin populations, and ampC mutations in the cefotaxime populations, I think

warrants mention.

Response 2: We sincerely appreciate the reviewers' comments. We acknowledge our misinterpretation of Ref. 35 (Stefan et al., *FEBS Letters*, 2003) and have removed the corresponding description in the revised version. Additionally, we recognize that our interpretation of Ref. 46 in the original manuscript (Iyer et al., *bioRxiv*, 2020) was incorrect. Furthermore, given that this paper has not been published, we have also removed the related discussion.

Reply to Reviewer #3

We would like to thank the reviewer for the constructive suggestion to improve our manuscript. We have revised the manuscript following the suggestion. Our response to the comment is shown below.

Comment 1: Regarding the new genetic information, I am surprised the authors did not make a little more about the information. Specifically, the fact that 'expected' mutations in the canonical resistance target, gyrA, were found in the ciprofloxacin populations, and ampC mutations in the cefotaxime populations, I think warrants mention.

Response 1: As the reviewers pointed out, providing information on the identified mutations in the evolved strain is expected to enhance the value of this paper. In response to this comment, we have added the following paragraph to the revised manuscript.

Revision 1 (Line 228):

Furthermore, this analysis demonstrated that mutations commonly associated with antibiotic resistance evolution are also prevalent in mutator populations. Under CFIX selection, mutations in *ampC*, which encodes β-lactamase, and *ompR*, which is involved in the regulation of porin protein, were detected in multiple evolved populations. These mutations are well-documented for their role in conferring resistance to β-lactams, including CFIX [36,37].

Similarly, in populations evolved under CP selection, mutations were frequently identified in *acrB*, which encodes a multidrug efflux pump, as well as in *acrR*, *marR*, and *soxR*, which regulate its expression. These mutations are also known to contribute to resistance against a broad spectrum of antimicrobial agents [38].
