# Supplementary material for: Quantitative analysis of relationship between mutation rate and speed of adaptation under antibiotic exposure in Escherichia coli
Source: PLoS Genet. 2025 Mar 28;21(3):e1011627. doi: 10.1371/journal.pgen.1011627 (PMC11975134; doi:10.1371/journal.pgen.1011627)
Supplement: Attachment — Submitted filename: Response_to_Reviewers.docx [file pgen.1011627.s011.docx]

**Reply to Reviewer #1**

**Comment 1-0**: The authors’ major conclusion is that there is an optimal mutation rate for the evolution of drug resistances and that a mutation rate higher than the optimum appears to inhibit that evolution. The authors’ simulations of the results lead to the interesting conclusion that high drug sensitivity minimizes the effect of mutation rate on the evolution of drug resistance because fewer cells with beneficial mutation survive. That is, what is important in determining the relationship between mutation rate and speed of evolution is the effective population size under selection. This conclusion will inform our understanding and help to direct further experimentation on the evolution of drug resistances

**Response 1-0**: We appreciate the reviewer's constructive suggestions for improving our manuscript. We have revised the manuscript accordingly and have detailed our point-by-point responses below.

**Major comments**

**Comment 1-1**: The main conclusion rests on the observation that the strain with the highest mutation rate failed to have the highest rate of evolution to drug resistances (Figs 3 & 5). But, that strain was a mutLdnaQ double, and it is clear from Fig S1B that the mutLdnaQ strain had a significantly lower growth rate than any other strain even in the absence of drugs. This result suggests that the strain is simply “sick” and that is at least part of the reason it failed to evolve drug resistance. While the authors attribute its growth defect to its high mutation rate, there is another good reason that it might be sick. dnaQ encodes the epsilon subunit of Pol III, the replicative DNA polymerase. Epsilon is part of the core and interacts with the polymerase subunit, alpha, and with theta, the third member of the core. Previous work has shown that interaction of alpha and epsilon is required for fully processive DNA synthesis (Studwell, and O’Donnell, 1990 JBC 265: 1171), and the high rate of deletion formation in dnaQ mutants may be due to these replication defects (Saveson & Lovett, 1997, Genetics 146:457). dnaQ null mutant strains rapidly acquire suppressor mutations that allow DNA replication to recover. The original identified suppressor was in dnaE, which encodes alpha (Lancy et al, 1989, J. Bact 171:5572), but other suppressors are possible, and they might have other phenotypic effects. Clearly, with the loss of MutL, the high mutation rate in the dnaQ mutant is further increased, but the data in this paper do not convincingly show that it is solely the high mutation rate, and not also the loss of other properties due to replication defects, that are responsible for the poor evolution of the mutLdnaQ stain.

**Response 1-1**: We greatly appreciate your insightful comments. As the reviewer pointed out, there are mechanisms of growth defects in mutators that are independent of the observed mutation rate. The deletion of *dnaQ* significantly increases replication defects and deletion formation (Saveson & Lovett, 1997, Genetics 146:457), which likely causes the observed growth defects. Additionally, the activation of post-replicative mismatch repair systems is suggested to contribute to slow growth in *dnaQ* silenced strains (Stefan et al., 2003, FEBS Letters 546:295). Quantifying the contribution of these factors to growth defects through mutation rates is difficult, as lethal deletions are not detected and mutations repaired by the post-replicative mismatch repair system are not observable in sequencing analysis.

It should be stressed that our analysis, utilizing a population dynamics model, incorporates these factors for the growth defects. The underlying assumption is that the contributions of such non-observed factors are proportional to the observed mutation rate. Under this assumption, the equation $\mu=\mu_{0}\exp\left( -\delta p \right)$, describes the dependency of population growth rate $\mu$on mutation rate $p$, incorporating both the effects of a high mutation rate and non-observable factors. Although the relationship between observed mutation rates and the contributions of non-observable factors to the growth defect may not be strictly linear and could follow non-linear functions, we have adopted a simplified assumption for clarity in this study.

The severe growth defect observed in *mutL*-*dnaQ* double deletion strains is attributed to the disruption of the post-replicative mismatch repair systems. In *dnaQ* mutant strains, it is suggested that the repair systems are activated; however, the deletion of *mutL* disrupts this system, potentially resulting in severe growth defects due to a high frequency of lethal mutations. Although such lethal mutations are not detectable by our sequence analysis, our population dynamics model accounts for this factor using the previously mentioned assumption.

In the revised manuscript, we have included additional discussions on the modeling of deleterious effects on growth rate, discussing the possible mechanisms of growth defects and our assumptions in the model. We are grateful for your comments, which have prompted us to enrich the manuscript with text that deepens the understanding of these phenomena.

**Revision 1-1** (page 8, Line 262):

Note that the observed reduction in growth rate may not be fully explained by high mutation rates alone. In fact, a previous study demonstrated that silencing *dnaQ* expression leads to a severe decrease in growth rate, a change that does not correspond to high mutation frequencies. Instead, this phenomenon is suggested to result from the recruitment of post-replicative mismatch repair systems [36]. Although the impact of such factors on growth defects cannot be quantified by mutation fixation rates, for simplification, we have assumed that the contributions of these non-observable factors are proportional to the observed mutation rate. Under this assumption, Eq. (2) describes the relationship between population growth rate and mutation rate, incorporating both the effects of high mutation rates and non-observable factors. We then used these fitted parameters for the numerical simulations.

**Revision 1-1** (page 14, Line 453):

These insights indicate that while a genetic load of deleterious or lethal mutations can partially explain the observed growth defects, other mechanisms are also likely involved. One such potential cause is the cost associated with high-frequency gene repair, which could deplete the cellular resources necessary for growth [25,35]. Since these mechanisms do not necessarily alter the genome sequence, assessing their contribution to the growth defect through mutation rate analysis alone is challenging

**Comment 1-2**: As shown in Fig. 1 and discussed in lines 137ff; the various strains have different mutational spectra. In particular, the MMR mutants enhance transitions, whereas the presence of the mutT deletion in any strain results in a high rate of AT to CG transversions. This is completely as expected from previous reports. What is striking is that in Fig. 3A&B the strains with the highest evolved drug resistances were mutT mutants (this is assuming I can correctly identify the symbols, some of which are nearly identical). This result suggests that these strains could become drug resistant not just because of their high mutation rate, but also because AT to CG transversions are more likely to give drug resistance than transitions. This possibility could be because the mutT mutants produce a greater proportion of nonsynonymous mutations than the other mutators (Table 1& S2).

**Response 1-2**: We apologize for the poor visibility of the markers in Fig.3. In the revised manuscript, we have improved the visibility of these markers.

In response to the reviewer’s comment, we analyzed the mutation spectrum of fixed mutations in antibiotic-resistant strains from a previous study. If A:T→C:G mutations led to a large number of non-synonymous substitutions, thereby contributing to the acquisition of drug resistance, an enrichment of A:T→C:G mutations in antibiotic-resistant strains would also be expected in the evolutionary dynamics of non-mutator strains. This was not consistently observed. We analyzed mutations in previously published data of *E. coli* experimental evolution under antibiotics (Suzuki et al, Nature Commun. 2014), summarized in Table S8. It appears that A:T→C:G mutations are not consistently enriched; rather, the distribution of mutations aligns with the base-pair substitution profile of wild-type *E. coli* (Lee et al., PNAS, 2012). This suggests that A:T→C:G mutations in *mutT* deletion strains do not necessarily increase the likelihood of resistance enhancement or accelerate the evolution of drug resistance as hypothesized.

The scope of our study is limited by the number of evolved strains, and the data does not support a statistical analysis of this hypothesis. Nonetheless, it presents an intriguing line of inquiry that warrants further investigation in future research.

**Revision 1-2** (page 13, Line 419):

As shown in Fig. 1, the results of MA experiment indicate that A:T→C:G mutations become predominant due to the deletion of the *mutT* gene. Such changes in the mutation spectrum could potentially affect the rate of evolution. Indeed, the evolved strains with a relatively high evolutionary rate, approximately a mutation rate of 0.1, are those lacking the *mutT* gene, as demonstrated in Fig. 3. The frequent occurrence of A:T→C:G mutations in these strains may contribute to their high evolutionary speeds. To explore this possibility, we analyzed fixed mutations in antibiotic-resistant E. coli strains obtained in a previous study of experimental evolution [39]. We hypothesized that if A:T→C:G mutations significantly contribute to resistance acquisition, an enrichment of these mutations in antibiotic-resistant strains would also be expected in the evolutionary dynamics of non-mutator strains. However, as shown in Table S8, A:T→C:G mutations are not consistently enriched; instead, the distribution of mutations aligns with the base-pair substitution profile of wild-type *E. coli* [26]. This suggests that AT to CG mutations in *mutT* deletion strains do not necessarily increase the likelihood of resistance enhancement or accelerate the evolution of drug resistance. Given the limited amount of data and the challenges associated with conducting statistical analyses, it is difficult to draw definitive conclusions from this study. Future research with sufficient data should be conducted to further explore the relationship between the mutational spectrum in mutators and the evolution of drug resistance.

**Comment 1-3-1**: The three strains with the lowest growth rates are mutL, mutLmutQ, and mutLmuH. This suggests that the mutH and dnaQ alleles were recombined into the mutL single mutant strain that had a growth defect. The sequences of the genetic manipulations are not described, but I tried to confirm this assumption by looking at the mutations labeled “ancestor” of the individual strains in Table S1. I found only an A335V mutant in malP common to all the strains with mutL, including mutTmutL, which did not appear to have a growth defect. So I cannot attribute the low growth rates solely to a pre-existing mutation in the mutL parent before all the other mutant alleles were recombined in, but there does appear to be a growth defect common to all the mutL strains but mutTmutL, suggesting the defect existed at some point in the mutL parent.

**Response 1-3-1**: In response to the reviewer's concerns, we have detailed the sequences of genetic manipulations used to construct the single- and double-gene knockout strains in Fig. S1 of the revised manuscript. This figure also includes common mutations between parent and progeny strains, along with their growth rates. As the reviewer highlighted, the *ΔmutL* strain and two of its derivative strains, *ΔmutLΔmutH* and *ΔmutLΔdnaQ*, exhibit relatively large growth defects. In contrast, the third derivative, *ΔmutLΔmutT*, does not display such a growth defect. Furthermore, we confirmed a consistent pattern of common mutations among *ΔmutL* and the three double-knockout strains; they share only the *ΔmutL* and malP(A335V) mutations. These findings do not support the hypothesis that the growth defects in the *ΔmutL*, *ΔmutLΔmutH*, and *ΔmutLΔdnaQ* strains were caused by mutations shared among them.

To provide information on the mutator strain construction, we added the following sentences to the revised manuscript:

**Revision 1-3-1 (page 5, Line 129)**:

The sequence of gene deletions during the construction of the mutator strains is summarized in Fig. S1.

**Figure S1.** Mutator Construction Procedure. Each box represents either a mutator strain or the wild-type strain. Arrows depict the lineage from parent to offspring in the construction of mutators. The list of mutations on the right details those shared between each parent-child pair (refer to Table S1 for the complete list of mutations). The values indicated in each strain box represent the growth rates (1/h; mean ± SD).

**Comment 1-3-2**: Related to this comment: what are the mutations labeled “ancestor”? If, for example, mutS was the starting strain into which mutH and mutT were placed, should not all the “ancestor” mutations in mutS also appear in mutSmutH and mutSmutT? I found only seven mutations common to mut S and mutSmutH, and only four of these were in mutSmutT. This is confusing and the reader should not have to examine the ancestor mutations to understand the genetic manipulations. The authors should describe the constructions more completely and define what the ancestor mutations are.

**Response 1-3-2**: The mutations labeled as "ancestor" in Table S1 were identified in a mutator strain immediately prior to their use in mutation accumulation (MA) experiments and experimental evolution under antibiotics. After genetic manipulations, each strain was cultured for various purposes, including creating frozen stocks and preparing genomic DNA samples, during which additional mutations could accumulate. Consequently, the ancestor mutations do not necessarily exhibit a consistent pattern between parent and offspring strains. For instance, some ancestor mutations identified in the *ΔmutS* single-gene knockout strain were absent in the *ΔmutSΔmutH* double-gene knockout strain.

To clarify this information, we have added the following sentences to the caption of Table S1 in the revised manuscript:

**Revision 1-3-2 (the caption of Table S1)**:

The mutations labeled as "ancestor" were identified in a mutator strain immediately prior to their use in mutation accumulation (MA) experiments and experimental evolution under antibiotics. After genetic manipulations, each strain was cultured for various purposes, including creating frozen stocks and preparing genomic DNA samples, during which additional mutations could accumulate. Consequently, the ancestor mutations do not necessarily exhibit a consistent pattern between parent and offspring strains. See Fig. S6 for the sequence of gene deletions during the construction of the mutator strains.

**Specific comments**

**Comment 1-4**: 57-58: According to the drift-barrier hypothesis articulated in Ref. 2, there is no mechanism for selection to adjust the mutation rate to promote evolution. So this statement is a bit extreme, although the subsequent discussion modifies it.

**Response 1-4**: We acknowledge that the drift-barrier hypothesis outlined in Reference 2 does not suggest that mutation rates are adjusted to facilitate evolution. Our original statement in lines 57-58 exceeded the scope of the discussion in Reference 2. Accordingly, based on the reviewer's suggestion, we have amended the manuscript.

**Revision 1-4** (page 3, Line 55):

Mutations exert a double-edged-sword influence on organisms, conferring either beneficial or deleterious effects [1], suggesting that mutation rates dynamically change over the course of evolution [1–3].

**Comment 1-5**: 154: see above about other possible contributions to decreased growth rates

**Response 1-5**: Following the reviewer's comments, we have incorporated the following explanation into the relevant paragraph:

**Revision 1-5** (page 6, Line 163):

We then quantified the growth rates of the mutator strains in M9 minimum medium without antibiotics. As depicted in Fig. S2B, the growth rate decreases with an increasing mutation rate, a pattern also observed in other studies [34]. This observed decrease in growth rate is thought to be caused, at least in part, by an increase in mutation rate. However, the potential contributions of other mechanisms, such as replication inhibition and activation of proofreading mechanisms, are also suggested [35].

**Comment 1-6**: Fig. S1b, Fig 3: It is very difficult to distinguish which strains are indicated by the open circles in these figures. Especially, the symbols for L and HT are nearly identical. The closed circles for SH and LQ also look nearly the same.

**Response 1-6**: Following the reviewers' comments, we revised Fig. 3 and S1b to enhance their visibility.

**Comment 1-7**: 179-182: This sentence is difficult to understand

**Response 1-7**: We apologize for the unclear sentence. It has been removed from the revised manuscript as it was not necessary for explaining the results, and this deletion improves the readability of the text.

**Comment 1-8**: 183-185: it would be clearer if the statement excepting the AMK results was first so that the reproducibility statement directly applied to the rest of the experiments.

**Response 1-8**: Following the reviewer’s suggestion, we revised the sentence as follows:

**Revision 1-8** (Page 7, Line 199):

To assess the reproducibility of our observations, we conducted a replicate experiment over a shorter period of five days. Except for AMK, which exhibited relatively small absolute values of MIC doubling rates, the results demonstrated clear correlations between the adaptation speeds in the 9-day and 5-day experiments (Fig. S4). This emphasizes the reproducibility of our adaptation speed estimates.

**Comment 1-9**: 193: It appears in Fig 3 D & E that the strain with the highest mutation rate, LQ, does have a lower average MIC doubling rate compared to the other mutant strains. The authors might supply some statistics to support this statement

**Response 1-9**: Following the reviewer’s suggestion, we investigated whether the MIC doubling rate of the LQ strain was significantly lower than that of other mutant strains. Please note that, following Reviewer #2's suggestion, we revised the method for calculating the MIC doubling rate. Instead of using the slope of linear fitting, we now calculate the MIC doubling rate based on the MIC change before and after experimental evolution. As a result of this modification, the MIC doubling rates presented in Fig. 3 have been slightly adjusted.

We selected five strains with mutation rates approximately 0.1 (namely T, HT, LT, ST, and QT strains) for comparison. Using the Mann–Whitney U test, we found significant differences in the MIC doubling rates of the LQ strain under CP and TP selections, rejecting the null hypothesis at a significance level of 0.01. However, under selections with AMK, CFIX, and CPFX, the null hypothesis of no difference could not be rejected.

The results of this statistical analysis substantiate the claims made in the original manuscript. To reflect these findings, the relevant section has been revised accordingly.

**Revision 1-9** (Page 7, Line 204):

Figure 3 illustrates the relationship between the mutation rate and the speed of adaptation for each antibiotic. As observed, these dependencies did not always follow a monotonic pattern. Notably, the LQ strain, which had the highest mutation rate, often showed slower adaptation speeds than strains with more moderate mutation rates. Moreover, the dependency on mutation rate varied with the antibiotics used for selection. For CP and TP selections, significant decreases in MIC doubling rates were observed in the LQ strain compared to the other five strains with mutation rates close to 0.1 (p<0.01; Mann-Whitney U-test). Conversely, no significant decreases were observed for the other three drugs.

**Comment 1-10**: 198ff: this reviewer found it easier to follow the mathematical modeling with a table defining the terms, but I still found it difficult. Note that δ is not defined.

**Response 1-10**: Following the reviewer's recommendation, we have incorporated Table 2 into the revised manuscript to summarize the variables and parameters used in the population dynamics model. We anticipate that the addition of this table will significantly enhance the readability of the modeling section of the paper.

**Table 2.** Summary of variables and parameters used in the population dynamics model.

**Comment 1-11**: 220-223: As discussed above, other factors could contribute to growth rate decline. In Fig S1B it is obvious that two strains, L and LH, have close to the same mutation rates as 4 other strains yet have significantly lower growth rates.

**Response 1-11**: As the reviewer pointed out, the *ΔmutL* and *ΔmutLΔmutH* strains exhibited lower growth rates compared to mutators with similar mutation rates, suggesting that the reduction in growth cannot be solely explained by mutation rates. Following the reviewer’s comments, as detailed in Response 1-1, we have expanded our discussion on mechanisms of growth defects that are not directly related to high mutation rates. Additionally, we have included an explanation about the assumptions underlying Eq. (2).

The mechanism underlying the slow growth observed in *mutL* deletion strains remains unclear. One possible explanation involves an imbalance between the functions of the MutL and MutS proteins. As reported (Acharyam, Mol. Cell 12, 233-246, 2003), MutL and MutS coordinately contribute to mismatch repair; MutS binds specifically to mismatched DNA, while MutL helps to release the bound MutS. This suggests that the balance between MutS and MutL proteins is crucial, which may be disrupted by the deletion of the *mutL* gene. The resulting imbalance between the amounts of MutS and MutL could lead to the observed growth defect. Indeed, the overexpression of MutS has been shown to cause cell division defects (Iyer, bioRxiv, 2020), which might support this hypothesis. Nonetheless, other potential mechanisms for the slow growth in *mutL* deletion strains should be explored in future studies.

In the revised manuscript, we have incorporated the above discussion regarding the observed slow growth in *mutL* deletion strains.

**Revision 1-11** (Page 14, Line 459):

The deletion strains of *mutL* exhibited lower growth rates compared to mutators with similar mutation rates (Fig. S2B), which also suggested that the reduction in growth cannot be solely explained by mutation rates. The mechanism underlying the slow growth observed in *mutL* deletion strains remains unclear. One possible explanation involves an imbalance between the functions of the MutL and MutS proteins. As reported [44], MutL and MutS coordinately contribute to mismatch repair; MutS binds specifically to mismatched DNA, while MutL helps to release the bound MutS. This suggests that the balance between MutS and MutL proteins is crucial, which may be disrupted by the deletion of the *mutL* gene. The resulting imbalance between the amounts of MutS and MutL could lead to the observed growth defect. Indeed, the overexpression of MutS has been shown to cause cell division defects [45], which might supports this hypothesis.

**Comment 1-12**: 290-294: It is not clear why molecular weight of the antibiotic should influence the beneficial effect of a mutation. More likely it is the mechanism of the antibiotic.

**Response 1-12**: We apologize for any ambiguity in this part. We can consider the following hypothesis regarding the mechanism by which the variable epsilon, representing the beneficial effect of mutation, correlates with the molecular weight of antibiotics: One of the main mechanisms for acquiring antibiotic resistance is the activation of efflux pumps. The activity of these pumps may depend on the molecular weight of the drugs, suggesting that the effects of mutations may also depend on molecular weight. While we currently lack data to support this, it has been presented as a possible hypothesis. To enhance clarity and readability, we have made the following revisions to the discussion:

**Revision 1-12** (Page 11, Line 342):

Regarding the observed correlation between $\epsilon$ values and the molecular weight of antibiotics, we propose the following hypothesis: One of the main mechanisms for acquiring antibiotic resistance involves the activation of efflux pumps. The activity of these pumps may be influenced by the molecular weight of the drugs, implying that mutation effects might similarly depend on molecular weight. Currently, there is no experimental data to substantiate this relationship; however, we believe these parameter estimates offer a novel perspective for further analysis.

**Comment 1-13-1**: The351ff: Lukacisinova et al conclude that more sensitive strains evolved resistance faster than more resistant strains. This seems to contradict the conclusions of this paper.

**Response 1-13**: As the reviewer has noted, the study by Lukacisinova et al. found that strains with higher susceptibility tend to evolve more rapidly. These results are not necessarily at odds with our own findings from evolutionary experiments using mutator strains. The accompanying figure plots the initial MIC against the increases observed in MIC over nine days of experimental evolution under five different antibiotics. Notably, the LQ strain is excluded from the plots as it sometimes did not exhibit a significant increase in MIC. Given our small sample size, establishing a statistically significant correlation through testing is challenging. However, there is an observable trend where strains with lower initial resistance tend to experience relatively larger increases in resistance. This observation suggests that our data do not contradict the findings reported by Lukacisinova et al.

**Figure 1-13.** Correlation between initial resistance (Day 0) and the increase in resistance after nine days of experimental evolution. Each point represents the observed relationship in mutator strains. To prevent data point overlap, small Gaussian noise (mean = 0, standard deviation = 0.1) has been added to both x and y coordinates. The Spearman’s rank correlation coefficient ρ is presented in each panel, calculated prior to noise addition.

**Comment 1-13-2**: Also, Lukacisinova et al argue that using strains without growth defects eliminates the possibility that selection would act on reversing the growth defect instead of antibiotic resistance. This is related to my concern, discussed above. The authors should discuss these differences in approach and conclusions.

**Response 1-13-2**: As the reviewer has highlighted, selection could potentially act on the growth defect often observed in the mutator strains utilized in our experimental evolution. To investigate this possibility, we assessed the growth rates of three strains (WT, *ΔmutS*, and *ΔmutLΔdnaQ*) both before and after experimental evolution. As presented in Fig. S9 of the revised manuscript, the growth rates remained largely unchanged. This strongly indicates that the observed increases in MIC during our experiments were not due to recovery in growth.

**Figure S9.** Changes in growth rate during resistance evolution. Growth rates for three strains (WT, S, and LQ) were quantified both before and after experimental evolution. For each selection drug, two independently evolved strains were analyzed. The bars represent the standard deviation of three replicate measurements.

**Revision 1-13-2** (Page 14, Line 470):

In the experimental evolution under antibiotics, there exists a possibility that selection may favor reversing growth defects in ancestral strains rather than enhancing antibiotic resistance [43]. To investigate this in our experimental evolution with mutator strains, we measured the growth rates of three strains (WT, S, and LQ) both before and after experimental evolution. As presented in Fig. S9, the growth rates remained largely unchanged. This finding strongly suggests that the observed increases in MIC during our experiments were not attributable to recovery in growth rates but were likely driven by adaptations related to antibiotic resistance.

**Comment 1-14**: 372-393: The authors should discuss the results I list in my first comment about dnaQ, viability and suppressors.

**Response 1-14**: Following review’s comment, the discussions related to comment 1-1 are incorporated in the Discussion part as follows.

**Revision 1-14** (Page 13, Line 437):

The relationship between mutation rate, fitness, and the evolution of drug resistance remains complex and not fully understood. In our mutator library, the LQ strain exhibited the highest mutation rate observed, approximately one base pair per genome per generation, and showed a significant reduction in growth rate (about 30% lower than the wild-type strain). While the higher mutation rate might contribute to this growth defect, the mechanism for the reduction in growth rate cannot solely be attributed to the genetic load caused by the high mutation rate itself.

For instance, research using microfluidics mutation accumulation (µMA) experiments by Robert et al. [44] revealed that approximately 1% of mutations are lethal, and 0.3% of the fixed mutations incur a fitness cost of over 30%. Assuming that 1.3% (the sum of 1% lethal and 0.3% costly mutations) of all mutations have nearly lethal fitness costs, the observed mutation rate in the LQ strain would only account for a 1.3% reduction in growth rate. Moreover, as previously discussed, silencing dnaQ expression results in a severe decrease in growth rate, a change not directly correlated with high mutation frequencies. This suggests that the reduction may result from the recruitment of post-replicative mismatch repair systems [35].

These insights indicate that while a genetic load of deleterious or lethal mutations can partially explain the observed growth defects, other mechanisms are also likely involved. One such potential cause is the cost associated with high-frequency gene repair, which could deplete the cellular resources necessary for growth [25,35]. Since these mechanisms do not necessarily alter the genome sequence, assessing their contribution to the growth defect through mutation rate analysis alone is challenging.

**Comment 1-15**: Table 1 and S2: A mean of 13.33 synonymous BPS appears 3 times, which seems unusual. Are these correct or was there a miscalculation or typo?

**Response 1-15**: We have confirmed that the strains SH, ST, and QT each accumulated a total of 40 mutations across three replicate lines by coincidence, which accounts for the average value of 13.33 mutations per line (calculated as 40/3).

**Comment 1-16**: 466: AT to CG doesn’t belong in this sentence

**Response 1-16**: Thank you for identifying the error. The term “A:T → C:G” has been removed from the text.

**Reply to Reviewer #2**

**Comment 2-0**: The manuscript submitted to PLoS Genetics by Shibai et al. reports on the relationship between mutation rate and adaptation rate under antibiotic selection. While the findings are interesting, they are not entirely novel, as previous studies have also shown that a high mutation rate can be costly and does not necessarily lead to better adaptation due to the potential impact on key processes such as replication, transcription, and translation. Although this is an interesting manuscript, it has several weaknesses that need to be addressed before it can be considered for publication.

**Response 2-0**: We appreciate the reviewer's constructive feedback and have revised our manuscript accordingly. Below, we detail our responses to each point raised. We believe that the revisions made to the manuscript adequately address your concerns.

We would like to highlight the novelty of this study. It is the first quantitative analysis exploring the relationship between mutation rate and the speed of adaptation across more than 10 mutator strains. A pivotal finding is the non-monotonic relationship between mutation rate and the speed of adaptive evolution, which is influenced by the mode of action of the drugs used in selection. This non-monotonic relationship was further elucidated using a simple population dynamics model. We believe that these analyses provide significant insights into the evolution of mutation rates.

**Major comments**

**Comment 2-1-1**: When calculating mutation rates, only single nucleotide polymorphisms (SNPs) are considered, which may be problematic. This approach overlooks several loss-of-function mutations, such as indels in a repressor of an efflux operon, that can lead to resistance.

**Response 2-1-1**: Following the reviewer’s suggestion, we recalculated the mutation rate by summing the identified single nucleotide polymorphisms (SNPs) and insertions/deletions (indels), and replotted the relationship between this mutation rate and the speed of evolutionary adaptation to antibiotics. This updated plot has been included in the revised manuscript as Fig. S5. The resulting relationship, characterized by a non-monotonic curve, is qualitatively identical to that depicted in the original Fig. 3. To incorporate this result, we have revised the following paragraphs to the revised manuscript:

**Revision 2-1-1** (Page 5, Line 138):

We calculated the mutation rates per generation for each strain following a previous study [29]. Initially, we determined the proportion of each pattern of synonymous base pair substitutions. Subsequently, we estimated the genome-wide mutation rate by dividing the number of accumulated synonymous mutations in a genome by the number of generations and then normalizing it with the frequency of possible mutational patterns. Additionally, the frequency of insertions and deletions (indels) was calculated.

**Revision 2-1-1** (Page 6, Line 204):

Figure 3 illustrates the relationship between the mutation rate and the rate of adaptation for each antibiotic. In this analysis, the mutation rate was calculated based on the frequency of SNPs. As observed, these dependencies did not always exhibit a monotonic pattern. A similar non-monotonic pattern is also observed when the mutation rate is calculated using the combined frequency of SNPs and indels (Fig. S5).

**Figure 1.** Mutation rates of mutator E. coli strains. Each dot with error bars represents the mean and standard deviation of the mutation rates observed in replicate MA lineages. The BPS rate (closed circle) was estimated using only synonymous mutations to exclude the effects of purifying selection. The indels rate (open circle) was also calculated only from the number of intergenic indels and normalized by dividing by the fraction of the length of the intergenic region in the genome (11%). The pie charts above the dots show the distribution of substitution patterns identified in the mutator strains, excluding the wild-type strain, which had as few as two mutations.

**Comment 2-1-2**: Additionally, mutation rates are calculated using only the final time points of evolved cultures, assuming that mutations accumulate monotonically. This assumption might be problematic and not consistent because the rate of adaptation is calculated using continuous phenotype-changing data over time (Figure 2, Figure S2).

**Response 2-1-2**: As the reviewer pointed out, our calculation of mutation rates is based on the assumption that mutations accumulate linearly during MA experiments. We acknowledge that in reality, the accumulation of mutations could be more dynamic and complex. However, due to the absence of time-series data on mutation accumulation, we adopt the straightforward assumption of a linear increase in the number of mutations over generations. This approach represents the least biased assumption given the limitations of our data size. Supporting this assumption, a study by Foster's group (Lee et al., PNAS, 2012, doi:10.1073/pnas.1210309109) demonstrated that the mutation rates for the first 3,000 generations were not significantly different from those over the entire span of 6,000 generations in their MA experiments. This finding aligns with our assumption that the number of mutations increases monotonically during our MA experiments. To discuss this assumption further, we have added the following sentences to the revised manuscript:

**Revision 2-1-2** (Page 5, Line 144):

In this analysis, the mutation rate was calculated based on the number of mutations observed at the endpoint of the MA experiments. This calculation assumes that the number of mutations accumulates linearly during the MA experiments. While the dynamics of mutation accumulation could potentially be more complex and variable, we adopt this simple assumption as it represents the least biased approach. Supporting this assumption, a previous study [26] demonstrated that during MA experiments spanning 6,000 generations, the mutation rates for the first 3,000 generations and the entire 6,000 generations were not significantly different. This observation is consistent with our assumption that the number of mutations increases monotonically during our MA experiments.

**Comment 2-2**: The rate of adaptation is calculated (Figure S2) by fitting a line to the log(MIC) versus time trajectories. This approach is problematic because adaptation functions can be monotonic, stepwise, or sigmoidal, leading to poor fits in many cases. A better algorithm, such as fitting a log-logistic function, should be used. Alternatively, to be consistent with how mutation rates are calculated, only the net change over 9 days (delta-MIC) can be considered.

**Response 2-2**: We appreciate the reviewer’s suggestion regarding the calculation of the rate of adaptation. We acknowledge that fitting a linear model to the log(MIC) versus time trajectories, as originally presented in Fig. S2, may not be optimal or problematic in cases where the adaptation pattern deviates from linearity.

To address this concern, as the reviewer suggested, we alternatively use the net change in MIC over the 9-day period (delta MIC). We calculated the net change in the log2-transformed MIC over the 9-day period and defined the speed of adaptation as the daily increase in log2-transformed MIC. We also confirmed that, except for AMK, this index shows reproducibility with the result of the preliminary 5-day experiment as same as in the case of the linear fitting shown in Fig. S4. To reflect these changes, we have modified Figs. S3, S4 and the corresponding part of the manuscript as follows:

**Revision 2-2** (Page 6, Line 191):

Using these time series MIC data, we evaluated the speed of drug resistance evolution under antibiotic treatment by determining the change in log_2_-transformed MIC per day, which was obtained by comparing the MIC values of the first and last days of the 9-day evolutionary experiment. We assumed that MIC directly corresponds to fitness, disregarding the non-monotonic relationship between MIC and fitness as well as the dependency of the MIC-fitness relationship on drug concentration.

**Comment 2-3**: The assumptions in the population dynamics model are overly simplistic and sometimes problematic. For example, a monotonic increase in the benefit function is assumed as bi=b0+i⋅ϵ. This assumption is difficult to justify in the context of antibiotic resistance evolution. For instance, a single gyrase mutation might be sufficient for bacteria to survive ciprofloxacin, with all subsequent mutations having negligible effects within the experimental range.

**Response 2-3**: In our population dynamics model, we have made several simplifying assumptions to facilitate appropriate parameter estimation. Although this simplification carries the risk of omitting critical elements necessary to fully explain the observed phenomena, it provides a powerful tool for the quantitative understanding of the evolving population.

The assumption $b_{i}=b_{0}+i\epsilon$ is grounded in observations from our nine-day experimental evolution under antibiotics, where the log-transformed minimum inhibitory concentration (MIC) increased approximately linearly over time across many culture lines. This observation suggests that a model where a single mutation exponentially increases the MIC is approximately suitable.

However, for some lineages, the log-transformed MIC shows a saturation curve, suggesting that mutations fixed later have a diminishing effect. This trend has been observed in several experimental evolution studies, including Lenski’s long-term experimental evolution (Barrick et al., Nature 2009). To account for this, we have modified our model to include a saturation curve for the increase in fitness due to mutations, as follows:

$b_{i}=b_{0}+{i\epsilon S}/{(i+S)}$

Here, $S$ is a parameter that influences the shape of the saturation curve. When $S$ is small, the increase in log-transformed MIC saturates with only a few mutations, whereas a larger $S$ results in an almost linear increase, akin to the original model.

Using this modified model, we estimated other parameters using a genetic algorithm. The accompanying figure illustrates how the average residuals between the model predictions and observed data points change when parameter $S$ is fixed. As depicted, the residuals decrease as $S$ increases. This result supports the appropriateness of using the assumption in the original model, which corresponds to the case of large S.

To discuss the validity of the assumption, we have added the following paragraph and Fig. S10 to the Discussion section in the revised manuscript:

**Revision 2-3** (Page 14, Line 478):

The experiments and simulations presented in this study encompass numerous limitations and unvalidated hypotheses. For instance, in our population dynamics model, we assumed $b_{i}=b_{0}+i\epsilon$, which means that the log-transformed MIC linearly increases with the number of beneficial mutations. This assumption is grounded in observations from our nine-day experimental evolution shown in Fig. S3, where the log-transformed MIC increased approximately linearly over time across many culture lines. However, for some lineages, the log-transformed MIC shows a saturation curve, indicating that mutations fixed later have a diminishing effect. This trend has been observed in several experimental evolution studies, including Lenski’s long-term experimental evolution [43]. To account for this, we have modified our model to include a saturation curve for the increase in fitness due to mutations, as $b_{i}=b_{0}+{i\epsilon S}/{(i+S)}$. Here, $S$ is a parameter that influences the shape of the saturation curve. When $S$ is small, the increase in log-transformed MIC saturates with only a few mutations, whereas a larger $S$ results in an almost linear increase, akin to the original model. Using this modified model, we estimated other parameters using a genetic algorithm. Figure S10 illustrates how the average residuals between the model predictions and observed data points change when parameter $S$ is fixed. As depicted, the residuals decrease as $S$ increases. This result supports the appropriateness of using the assumption in the original model, which corresponds to the case of large $S$.

**Figure S10.** Relationship between the parameter $S$ and the residual of fitting. The parameter $S$, which describes the saturation effect of mutation accumulation in the form $b_{i}=b_{0}+{i\epsilon S}/{(i+S)}$, is plotted against the residual of the fitting. The modified model incorporating the saturation effect was used to fit the experimental data shown in Fig. 3. As observed, the residuals decrease with increasing $S$, suggesting that the saturation effect is negligible.

**Comment 2-4**: The authors state, "Moreover, we showed that the relationship between the mutation rate and speed of adaptation is contingent upon the mode of action of the drug involved." However, I am not convinced that this relationship is adequately explained, and I am concerned that the issues highlighted in points #1 and #2 may mislead the authors' conclusions.

**Response 2-4**: As outlined in Responses 2-1 and 2-2, we have addressed Comments 1 and 2 by including indels in our mutation rate calculations and by redefining the speed of resistance evolution as the net change in minimum inhibitory concentration (MIC) over the nine-day period (delta MIC). We observed that these modifications did not alter the relationship between mutation rate and speed of adaptation, demonstrating the robustness of our results. Additionally, in response to the reviewer’s suggestion, we examined whether the fit of our data would change by modifying the population dynamics model to incorporate a saturation effect in the MIC increase due to mutations (Response 2-3). We found that the results remained qualitatively unchanged, further underscoring the robustness of our findings. We believe that our conclusions regarding the non-monotonic relationship between mutation rate and speed of adaptation are convincingly supported by these analyses.

**Comment 2-5**: The claim that growth rates decrease as mutation rates increase is based on the fit in Figure S1, which is unusual and heavily influenced by a single data point.

**Response 2-5**: As the reviewer highlights, our data includes one mutator strain (LQ) with a notably reduced growth rate, which significantly influences the fitting. It is logically expected and supported by previous research that an increase in mutation rate can lead to growth defects. For instance, as depicted in the figure below, Ishizawa et al. (Genes to Cells, 20(1), 68-76, 2015) report a similar observation, despite using a different method for measuring mutation rates—estimating it through the frequency of drug-resistant strains. In their study, the relationship between mutation rate and growth rate is plotted across three different culture environments. The environment most similar to ours is the “mM63+AA”, corresponding to modified M63 minimum medium with supplementation of 20 amino acids. As demonstrated in their findings, and consistent with our observation in Fig. S1B, the growth rate decreases as the mutation rate increases.

**Figure 2 in Ishizawa et al.** Relationship between mutation rates and growth rates. In this study, mutator strains were created using single and double deletion of *mut* genes.

In the revised version of the paper, we added the following sentence to clarify that the observed decrease in growth rate due to an increased mutation rate has also been documented in other studies:

**Revision 2-5** (Page 6, Line 163):

We quantified the growth rates of the mutator strains in M9 minimum medium without antibiotics. As depicted in Fig. S1B, the growth rate decreases with an increasing mutation rate, a pattern also observed in other studies [34].

**Comment 2-6**: Some of the nomenclature is confusing. For example, what is meant by "mutS mutant"?

**Response 2-6**: Thank you for pointing out the use of inappropriate nomenclature. We have revised the sentence that included 'mutS mutant' accordingly. Additionally, we have reviewed the manuscript for any other instances of inappropriate nomenclature.

**Revision 2-6** (Page 4, Line 104):

A recent study by Gifford et al. examined the advantage of a mutator sub-population for the evolution of drug resistance [20]. They changed the frequency of the mutator strain in the evolving populations while using the same mutator strain made by *mutS* deletion for all populations, which might cause biases on the mutation spectra [21].

**Reply to Reviewer #3**

**Comment 3-0**: In this work, the authors have investigated the role of different mutation rates in resistance to five antibiotics. There is a lot I like about this study from an experimental point of view. In particular, I appreciate that the authors performed mutation accumulation to measure the relative mutation rates of the strains. I also like the combination of experiments with parameter estimation.

**Response 3-0**: We appreciate the reviewer's constructive suggestions for improving our manuscript. We have revised the manuscript accordingly and have detailed our point-by-point responses below.

**Major comments**

**Comment 3-1**: I was a little disappointed to not see any genetic data from the evolved resistant populations. Particularly, looking at the mutational spectrum from the MA experiments in the different mutators would nicely setup looking at how spectra affects the propensity to evolve different genetic bases for resistance—both in terms of specific mutations within resistance genes, but also the set of genes that acquire mutations. I really think this is a missed opportunity here that would greatly increase the impact of the study.

**Response 3-1**: Following the reviewer’s suggestion, we sequenced the genomes of evolved resistant populations to determine if the profile of mutated genes varies depending on the mutation spectra. We selected 24 populations after nine days of antibiotic selection, involving three mutator strains (deletion strains of *mutL*, *dnaQ*, and *mutT*), two antibiotics (CP and CFIX), and four independently evolved populations each. The mutations identified are summarized in Table S7. We observed that the mutation spectra of these fixed mutations align with those seen in mutation accumulation experiments (Fig. S6 in the revised manuscript; shown below), for example, A:T to C:G mutations were significantly enriched in the *mutT* deletion strain. Additionally, we noted that the mutator strains often targeted the same genes, such as *acrB/acrR* and *soxR* for CP and *ompR* for CFIX, to develop resistance to antibiotics. These findings imply that for each target gene associated with antibiotic resistance, there are multiple potential resistance-conferring mutations that *E. coli* can exploit based on their mutational patterns. While there could be some bias in the relationship between resistance acquisition and mutation spectra, our current dataset is too small to reliably detect such biases.

In the revised version of the paper, we have added the following sentences to present the mutation profile of the resistant populations:

**Figure S6.** Mutation Spectra in Resistant Strains. The distribution of substitution patterns and indels calculated by 24 resistant strains (three ancestor mutators, two antibiotics, and four replicates) is presented. Each dot represents the mutation fixation rate during the experimental evolution calculated based on SNPs (closed circle) or indels (open circle).

**Revision 3-3-2** (Page 7, Line 220):

To examine whether the profiles of mutated genes differ depending on the mutation spectra, we sequenced the genomes of evolved resistant populations. We selected 24 populations after nine days of antibiotic selection, involving three mutator strains (L, Q, and T), two antibiotics (CP and CFIX), and four independently evolved populations each. The mutations fixed in the populations are summarized in Table S7. We observed that the mutation spectra of these fixed mutations align with those seen in mutation accumulation experiments (Fig. S6), for example, A:T to C:G mutations were significantly enriched in the *mutT* deletion strain. Additionally, we noted that the mutator strains often targeted the same genes, such as *acrB/acrR* and *soxR* for CP and *ompR* for CFIX, to develop resistance to antibiotics. These findings imply that for each target gene associated with antibiotic resistance, there are multiple potential resistance-conferring mutations that *E. coli* can exploit based on their mutational patterns.

**Comment 3-2**: I would like to have seen some discussion about the epistasis between the mutator alleles themselves. To a first approximation, there are 4 mutation rates here (see Fig3), (1) wild-type (one point), (2) loss of function (LOF) in MutSHL or DnaQ alone (four points), (3) LOF of MutT with or without LOF of MutSHL (six points), and (4) combined LOF of both Mut(SH)L and DnaQ (one point). Changes in MIC were also more or less similar within these clusters.

Joint LOF of DnaQ+Mut(SH)L is clearly an oddity. Given this, it might be an idea to temper the conclusions on the lack of monotonicity, as it’s hard to know whether the evolution of MIC is indeed constrained at higher mutation rates (putatively by the accumulation of deleterious mutations) or because of some synthetically deleterious combination of these alleles. If the authors have this data, it would be nice to compare what the growth rates of the ancestral knockout strains are relative to wild-type, to see if there’s any big defect present already in the LQ strain (or indeed any of the strains). An absence of an initial growth defect would help strengthen the conclusion regarding the non-monotonicity of MIC with mutation rate. The presence of a growth defect would potentially complicate this.

**Response 3-2**: To demonstrate the epistatic effects among mutator alleles, we detail the sequences of genetic manipulations used to construct both single- and double-gene knockout strains in Fig. S1 of the revised manuscript. This figure illustrates that the reduction in growth rate from WT is greater in the double-gene knockout of mutL and dnaQ than the combined decreases observed in the individual mutL and dnaQ knockout strains. This suggests a synergistic relationship between their deleterious effects.

The observed growth defects could significantly influence the evolution of resistance, complicating the interpretation of our results. However, our population dynamics model, outlined in Eqs. (1) and (2) and accounting for mutation-rate dependent growth defects, successfully replicates the observed behavior in the experimental evolution, including the differing adaptation dynamics to bacteriostatic and bactericidal antibiotics. Despite the growth defects in mutator strains affecting resistance evolution, our analysis provides novel insights into the co-evolution of mutation rate and antibiotic resistance.

**Comment 3-3**: The authors may consider citing a paper from earlier this year by Elgrail et al. (2024, J Evol Biol) on ‘multi-locus mutators’, as it could provide additional support and context for their approach of studying the double knockout strains here. Pubmed link: https://pubmed.ncbi.nlm.nih.gov/38367184/

**Response 3-3**: Thank you for sharing this significant paper. We have referenced it in two sections within the introduction as follows:

**Revision 3-3-1** (Page 3, Line 63):

In another example, an antibiotic selection experiment involved a mutator *E. coli* strain that initially exhibited an increased mutation rate due to the knockout of one gene. Subsequent increases in the mutation rate, caused by mutations in additional genes, were repeatedly observed [7].

**Revision 3-3-2** (Page 4, Line 108):

To avoid capturing biased trends, we constructed 12 *E. coli* mutator strains with elevated mutation rates by deleting genes with different mutation spectra and combinations of those genes. It is important to note that the emergence of such multilocus mutators has been previously observed in antibiotic selection experiments [7].

**Comment 3-4**: If I had to find something to comment negatively about, for balance, one area that could be improved was the choice of antibiotics. The authors could alleviate this by mentioning that their study system is for understanding the general principles of resistance evolution, and not specific to particular clinical outcomes. While resistance to each of these can be conferred by spontaneous mutation, mutation isn’t the predominant mechanism of resistance for four out of five of them, outside of laboratory settings. Enzymatic resistance is more relevant in the clinical context for trimethoprim (via acquired dfrA genes), amikacin (aminoglycoside-modifying enzymes), chloramphenicol (chloramphenicol acetyltransferases) and cefixime (beta-lactamases). Ciprofloxacin resistance is, however, acquired by predominantly by mutation. This might not matter for the general readership of PLOS Genetics, but does detract from the applicability to real-world resistance for these particular drugs. To be fair to the authors, they are not explicitly claiming clinical relevance, but there is a tendency toward that thinking implicitly when resistance is studied.

**Response 3-4**: Thank you for your insightful comments. We agree that the simple mutations, such as SNPs and short insertions/deletions discussed in this study, are not the primary drivers of antibiotic resistance observed in clinical settings. However, the primary focus of our paper is on fundamental science, specifically aimed at elucidating mutation rates and speeds of adaptation during the evolutionary process. We use the evolution of antibiotic resistance primarily as a framework to analyze these aspects.

We recognize the potential future contribution of this basic understanding to medical research, which prompted a brief mention of medical applications in the text. However, your feedback highlights that this may have inadvertently suggested that our study directly addresses real-world antibiotic resistance. To prevent such misunderstandings, we have revised the manuscript to remove any statements that imply direct contributions to clinical applications.

**Revision 3-4-1** (original; Page 4, Line 116):

This study provides valuable insights into the mechanisms underlying the evolution of antibiotic resistance and highlights the importance of considering mutation rates when evaluating the efficacy of antibiotic treatments.

**Revision 3-4-2** (revised; Page 4, Line 118):

These findings offer important insights into the evolution of mutation rates accompanying evolutionary processes.

**Technical questions:**

**Comment 3-5**: Why was 34°C used for growth? This is not necessarily problematic, but it differs from others who have largely done experiments at 30°C or 37°C, and this introduces some difficulty when comparing with previous work. Growth temperatures can also affect mutation rates. A brief mention of why might put the readers at ease.

**Response 3-5**: The cultivation temperature of 34°C was chosen to align with our previous studies on the experimental evolution of *E. coli*, where this temperature was also used.

https://pubmed.ncbi.nlm.nih.gov/33235191/

https://pubmed.ncbi.nlm.nih.gov/25517437/

This specific temperature was chosen in those studies to moderately slow down the growth rate, which allows for the transfer of cells during the exponential growth phase in our experimental settings. To clarify this methodological choice, we have included the following explanation in the manuscript:

**Revision 3-5** (Page 18, Line 596):

Cultivation at 34°C was chosen to reduce the growth rate compared to the standard condition of 37°C, thereby facilitating subculturing during the exponential growth phase in our experimental settings.

**Comment 3-6**: Minimal medium with amino acids: Again, no issues with this, but a note about why this medium might be helpful. Particularly, it might help the field to know that rich media such as LB inhibit the effects of some antibiotics e.g. trimethoprim.

**Response 3-6**: To enhance the reproducibility of cultures and facilitate future analysis of medium components during resistance evolution, we opted for a synthetic medium with defined components rather than a rich medium like LB, which contains unspecified components. Moreover, to improve the robustness of *E. coli* against mutations (Ishizawa, Genes to Cells, 20: 68-76, 2015), we employed a minimal medium supplemented with amino acids. These revisions have been incorporated into the manuscript to clarify our rationale.

**Revision 3-6** (Page 16, Line 528):

In this study, to enhance the reproducibility of cultures and facilitate future analysis of medium components during resistance evolution, we opted for a synthetic medium with defined components rather than a rich medium like LB, which contains unspecified components. Moreover, to improve the robustness of *E. coli* against mutations [50], we employed a minimal medium supplemented with amino acids.

**Comment 3-7**: Insertion sequence (IS)-free E. coli K-12: I don’t think this is a problem, but could the authors please elaborate why they chose to use this interesting strain? I think they have done this because they want to study the pure effects of the mutator alleles. However, IS mobilisation plays an important role in resistance evolution by loss of function, both in laboratory and clinical strains. It would help the reader understanding.

**Response 3-7**: We agree that IS transposition significantly influences bacterial evolution. However, for this study, we selected an IS element-free strain to ensure reliable resequencing analysis. This decision was driven by the challenges associated with accurately determining the positions of IS element insertions using short-read sequencers. We have incorporated these revisions into the manuscript to further clarify our rationale.

**Revision 3-7** (Page 16, Line 519):

In this study, we utilized an IS-free strain to ensure reliable resequencing analysis, as determining the precise positions of IS element insertions can be challenging when using short-read sequencing technologies.

**Presentation comments:**

**Comment 3-8**: Fig1: I like this figure. I assume the pie charts are mutational spectra from the MA experiment, but this isn’t 100% explicit. Might it be helpful in the legend to group the mutations by transition/transversion?

**Response 3-8**: Following the reviewer’s suggestion, we have revised Fig. 1 to visually differentiate mutations by transition and transversion, providing clearer representation of the data.

**Figure 1.** Mutation rates of mutator E. coli strains. Each dot with error bars represents the mean and standard deviation of the mutation rates observed in replicate MA lineages. The BPS rate (closed circle) was estimated using only synonymous mutations to exclude the effects of purifying selection. The indels rate (open circle) was also calculated only from the number of intergenic indels and normalized by dividing by the fraction of the length of the intergenic region in the genome (11%). The pie charts above the dots show the distribution of substitution patterns identified in the mutator strains, excluding the wild-type strain, which had as few as two mutations.

**Comment 3-9**: Fig3: I find the colours palette non-intuitive and difficult to distinguish, in particular L/HT (both open points with a blue/green colour), and S/QT (two shades of yellow). The open-yellow Q points are also very hard to see. I can’t make out why some treatments were assigned open points.

I would suggest two things. First, use shapes in addition to colours. For instance, black squares for WT, coloured circles for the single-locus mutators (H,L,S,Q,T) and then coloured triangles for the two-locus mutators (LH,SH, LQ, etc…).

Second, group the labels according to the ‘clusters’ they form on the x-axis. If you group the labels in this way, then you only need to take care that the colours are very distinct within these. There are some nice options for six-colour palettes here: https://coolors.co/palettes/popular/6%20colors

FigS1: Repeat the colour scale legend here as it’s hard for the reader to make them flip between documents to read the figures, especially as it’s the same complicated colour scale as in Fig3.

**Response 3-9**: Thank you for your valuable suggestions. Following your advice, we have replaced all markers and colors in Fig. 3 and Fig. S1. We believe that these changes have significantly improved the readability of these figures.
